# Direct Conversion of Methane to Propylene

**DOI:** 10.34133/research.0218

**Published:** 2023-09-08

**Authors:** Yunpeng Hou, Yuxiang Lan, Chao Qian, Shaodong Zhou

**Affiliations:** ^1^ College of Chemical and Biological Engineering, Zhejiang Provincial Key Laboratory of Advanced Chemical Engineering Manufacture Technology, Zhejiang University, 310027 Hangzhou, P. R. China.; ^2^ Zhejiang Provincial Innovation Center of Advanced Chemicals Technology, Institute of Zhejiang University-Quzhou, 324000 Quzhou, P. R. China.

## Abstract

Nonoxidative coupling of methane exhibits promising prospect in that it affords value-added hydrocarbons and hydrogen with high atom economy. However, challenge remains in direct, selective conversion of methane to more valuable hydrocarbons like olefins. The current work presents a catalyst with well-dispersed Ta atoms anchored by graphitic C_3_N_4_-supported phthalocyanine. Such a catalyst is able to convert methane selectively to ethylene and propylene at a relatively low temperature (350 °C). The conception of the active center and construction of the catalyst have been described, and the origins of the catalytic performance are discussed.

## Introduction

The growing availability of low-cost and abundantly sourced natural gas leads to increased interest in its conversion to value-added chemicals. Natural gas is composed of dominantly small hydrocarbons with methane taking typically a volumetric fraction of about 70 to 90% [1–3]. Nowadays, great efforts have been conducted to convert methane into more useful chemicals like syngas, methanol, light olefins, aromatic compounds, etc. via direct or indirect routs [4]. The indirect rout involving the methane reforming and Fischer–Tropsch processes plays a crucial role in industry, as it affords one of the most important classes of chemicals—olefins [5]. However, such a 2-step conversion sequence wastes considerable part of methane molecules by unavoidably producing useless CO_2_ and H_2_O. By contrast, direct methane conversion shortens the reaction paths and utilizes more proportion of methane [6–7]. In general, there are 2 major routes for the direct conversion of methane to light olefins, i.e., oxidative coupling of methane (OCM) and nonoxidative coupling of methane (NOCM) [4]. The OCM process uses oxidant to overcome the thermodynamic restrictions and make the reaction exothermic [8]. However, the by-products like CO_2_ and H_2_O are still unavoidable, decreasing the atom economy.

Since 1990s, numerous efforts have been made to produce hydrocarbons through NOCM processes and promising progress achieved recently [9–11]. For example, a Pt–Bi bimetallic catalyst was reported to selectively convert methane to ethane with high carbon selectivity (>90%) and typical methane conversion of ca. 2% at 600 to 700 °C [9]. The Fe/SiO_2_ catalyst is capable of 48% methane conversion via the NOCM at 950 °C, producing ethylene, naphthalene, and benzene with a selectivity of 53%, 25%, and 22%, respectively [12]. Similar products over a Pt–Sn catalyst at 700 °C were observed, whereas the methane conversion was lower than 0.3% [13]. In addition, in NOCM, methane conversion may proceed at low temperatures: 6 wt% of Pt/SiO_2_ catalyst was reported to convert methane to hydrogen and C_2_H_6_ continuously at 250 °C [14]. Here, obviously, more efforts are required in the production of more valuable hydrocarbons like olefins via NOCM at low temperatures.

In 1974, it was reported that tantalum can convert methane or acetylene to carbon and hydrogen at 1,500 to 2,300 °C [15], kicking off the story of methane activation at the tantalum center. Later in 1991, the gas-phase activation of methane by Ta^2+^ ions was reported [16], followed by a series of gas-phase studies on methane activation by tantalum-involved ions [17]. A series of ions like [TaO]^+^ [18], [TaO_2_]^+^ [19], [TaO_3_]^+^ [20], [TaN]^+^ [21], and [TaCO_4_]^+^ [22] are able to break the H_3_C–H bond in the gas phase under ambient conditions. Here, the relativistic effects induced strong Ta–C interaction matters [23–25]. The excellent performance of Ta in gas-phase methane activation systems was later applied in the condensed phase: By building a H–Ta–O_2_ active site on silica, the catalyst converts methane to ethane with 98% selectivity at temperatures below 500 °C, although the methane conversion was below 0.5% [26]. The current work was also inspired by previous publications on gas-phase methane activation as mediated by Ta-involved species.

Notably, although Ta–O is an ideal center for methane activation, further conversion of the strong Ta–C bond may encounter high energy barrier [19–20]. To weaken the so-formed Ta–C bond, an electron-rich Ta center is required. This is supported by previous report on highly efficient thermal activation of methane by [TaN]^+^ in the gas phase [21]. Thus, in general, a Ta–N unit is preferable for methane conversion, and a Ta–N_4_ center may possibly be built in the condensed phase. Here, we report a catalyst with Ta–N_4_ center as anchored in phthalocyanine that is supported by graphitic carbon nitride (g-C_3_N_4_). Such a catalyst is able to convert methane selectively to propylene at a relatively low temperature (350 °C).

## Results and Discussion

As the participation of oxygen in the transformation of methane is not wanted, we decided to construct an active Ta–N_x_ center on the carbon-based materials. Thus, inspired by the synthesis method to prepare single atom catalyst [27–33]. Quite a few organonitrogen species were used to prepare Ta-complex precursors for further pyrolysis on carbon-based materials. However, the attempts with ethylenediamine, 2-methylimidazole, and 1,10-phenanthroline failed to produce an active catalyst. This was attributed to the lack of regulated coordination pattern all through the preparation of the supported catalyst. Thus, we tried to construct a macrocyclic ligand anchored Ta center, which was further supported on carbon nitride.

The structure of tantalum phthalocyanine (TaPc) with a Ta–N_4_ center was first reported in 1988 [34], and it has shown promising in electrocatalysis, particularly the oxygen reduction reaction [24,35–38]. TaPc was thus selected as the precursor complex, which was prepared according to previous reports [34]. In fact, we first tried thermocatalytic conversion of methane with TaPc only (confirmed by matrix-assisted laser desorption ionization mass spectrometry method; Fig. S1). As a result, trace product was observed at a temperature up to 450 °C. Considering that the TaPc molecules may stack upon crystallization [39–42], the unsatisfying performance of TaPc was attributed to low dispersity. A proper support is thus necessary to anchor each Ta–N_4_ center separately.

g-C_3_N_4_ has proven to be an ideal catalytic material for various chemical processes [43]. With the following advantages, g-C_3_N_4_ was selected as the active center carrier in NOCM: (a) It is usually prepared above 500 °C through tubular furnace with high thermostability; (b) g-C_3_N_4_ is of 2-dimensional structure and high electron mobility, and it can support a structure like TaPc stably via π–π stacking [44–45]; and (c) the nitrogen atoms of heptazine rings and the structural cavities may serve as ideal coordination sites to stabilize a metal center like Ta [46–47].

**Fig. 1.**
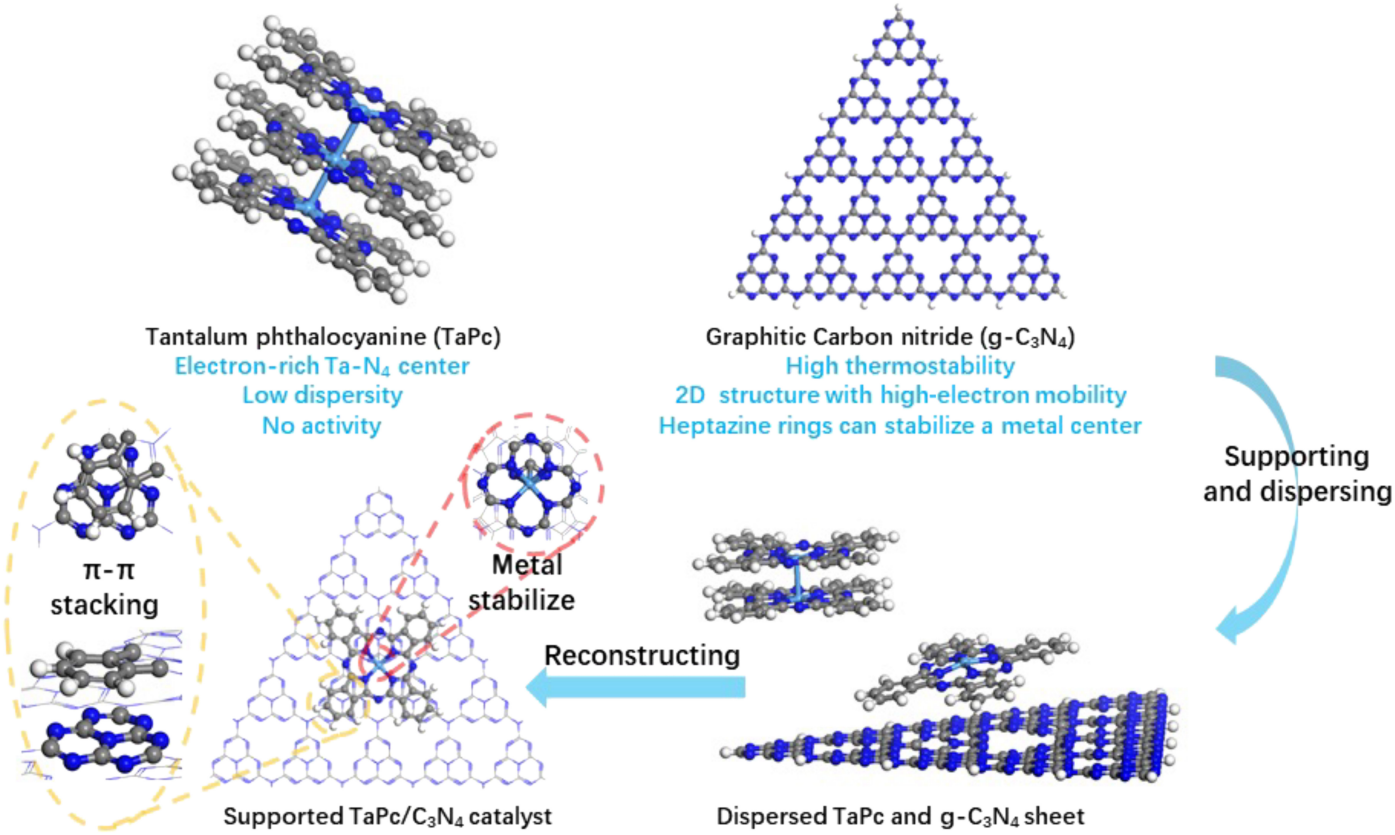
Schematic diagram of TaPc/C_3_N_4_ catalyst preparation.

**Fig. 2.**
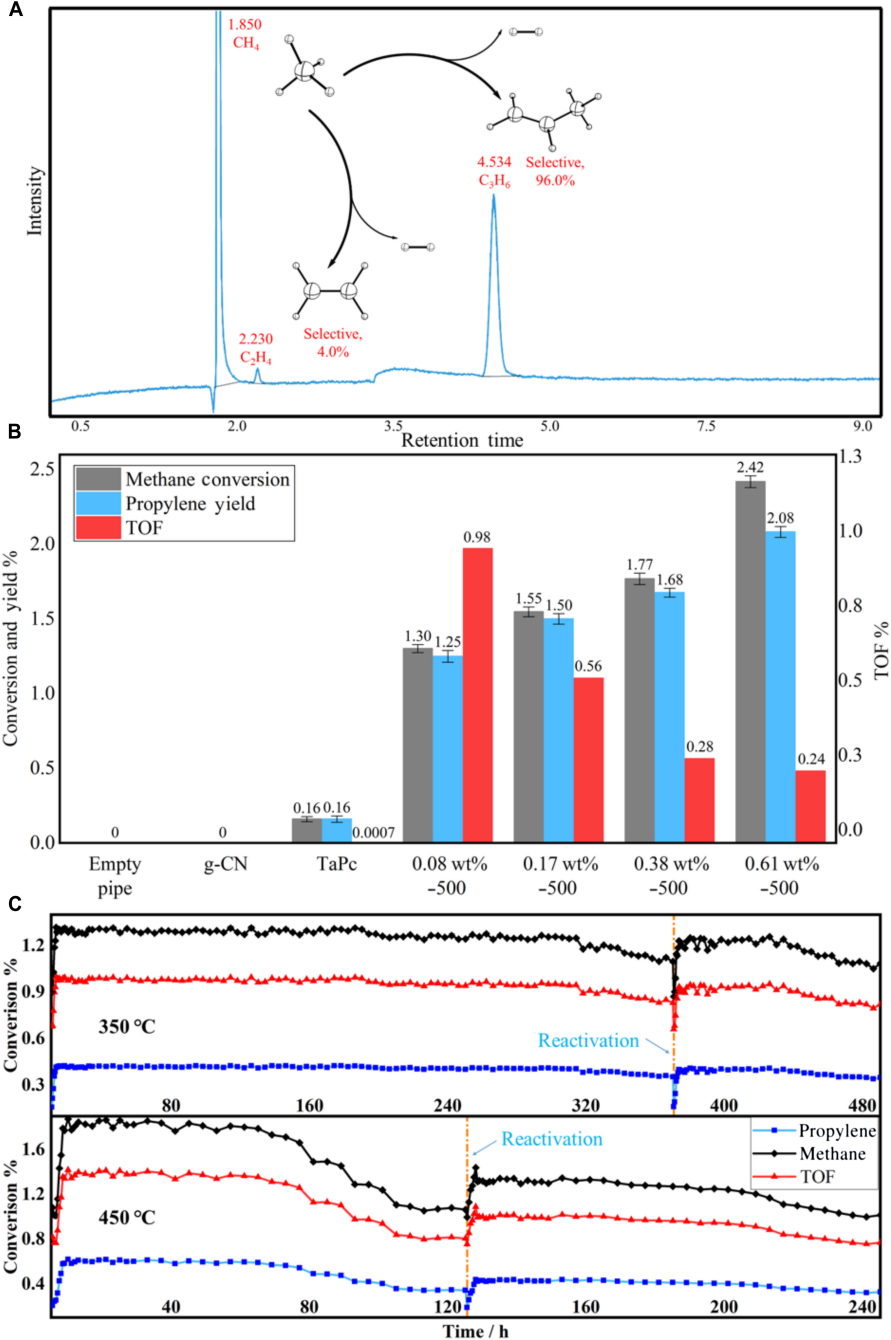
Performance of TaPc/C_3_N_4_ catalysts (A) the gas chromatography spectrum of TaPc/C_3_N_4_ (0.08 wt%) at 4 h in 350 °C. (B) The relationship of metal contains (Ta, wt%), propylene yield and TOF of TaPc/C_3_N_4_ catalysts after 4 h in 350 °C. (C) Lifetime of TaPc/C_3_N_4_ (0.08 wt%) at 350 and 450 °C, including reactivation, TOF, s^−1^.

After a series of attempts to support TaPc well on g-C_3_N_4_, the controlled sequential impregnation and pyrolysis procedures were found optimal. Through activation under hydrogen flow, the so-prepared TaPc/C_3_N_4_ catalysts exhibit surprisingly high activity toward methane conversion (Fig. 1). Moreover, highly selective generation of propylene was observed, and ethylene was produced as well. The performances of different TaPc/C_3_N_4_ catalysts are shown in Fig. 2. As shown in Fig. 2B, the higher the Ta content is, the higher activity the TaPc/C_3_N_4_ catalysts give. On the other hand, however, the yield of propylene is not increasing proportionally with the content of Ta, resulting in lower turnover frequency (TOF) values with over high Ta content. Thus, 0.08 wt% of Ta sample gives the highest TOF with the valve of 0.99 s^−1^ at 350 °C (Fig. 2B and Fig. S9B). At this condition, the selectivity of propylene is up to 96.0%, corresponding to 4.0% ethylene (Fig. 2A). Further, in the lifetime test, 0.08 wt% of Ta sample affords a long single-run lifetime of >300 h at 350 °C with stable conversion of methane (Fig. 2C); after reactivation, it still lasts for >120 h. Although higher TOF was obtained when performing the reaction at 450 °C, the lifetime of the catalyst is shortened dramatically (Fig. 2C). More technical details are provided in the Supplementary Materials (Fig. S9).

As there is no previous report on the production of propylene via NOCM, the products have been verified carefully: The produced hydrogen was detected through online gas chromatography (Fig. S2); the produced propylene and ethylene was first confirmed using gas chromatography upon comparison with standard samples, and further evidence was provided by gas chromatography-mass spectrometry (Fig. S3).

**Fig. 3.**
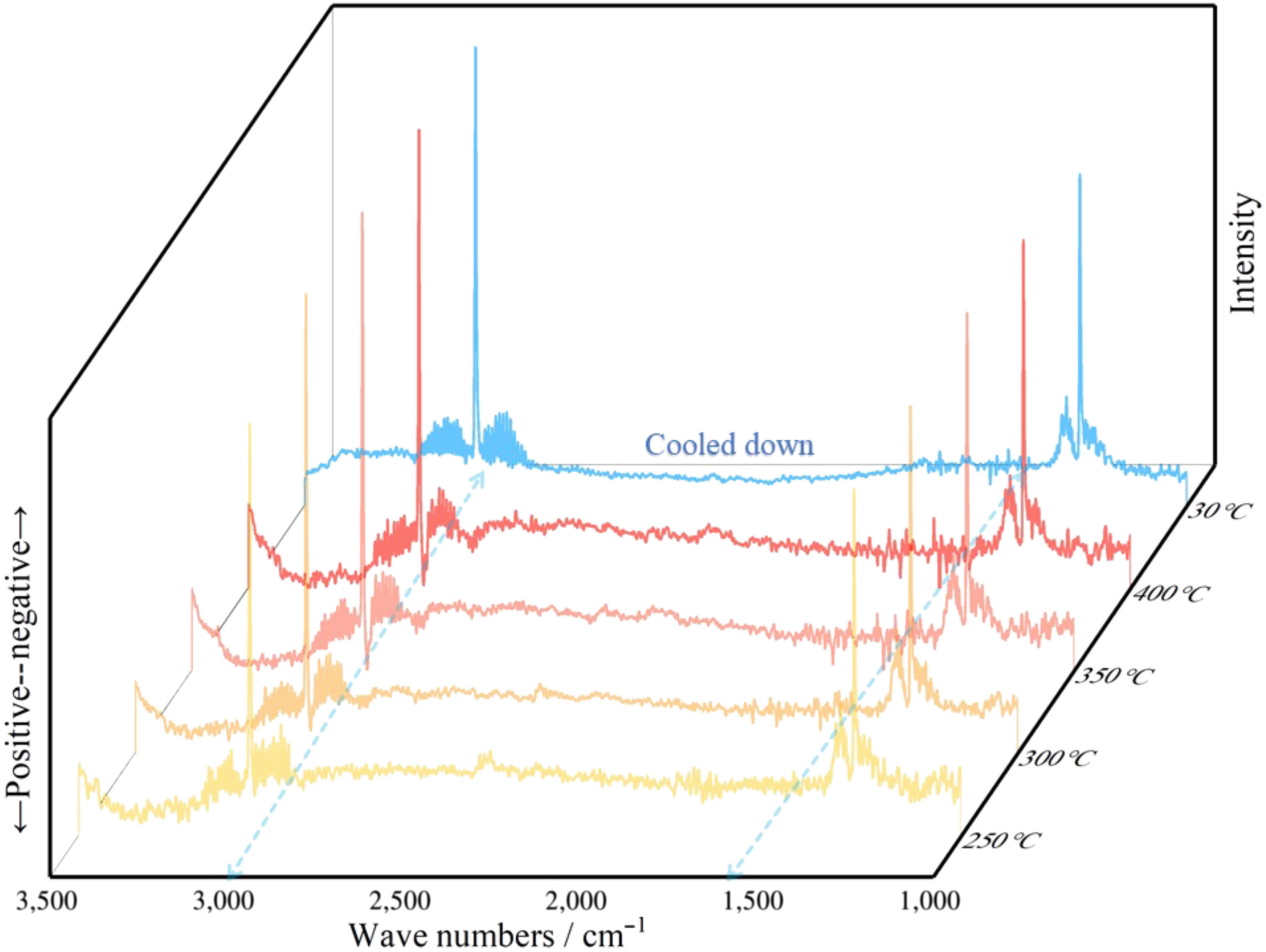
In situ diffuse reflectance infrared Fourier transform spectroscopy with temperature gradient of TaPc/C_3_N_4_ (0.08 wt%).

Further, the in situ diffuse reflectance infrared Fourier transform spectroscopy analysis was performed to obtain even more information regarding the methane conversion processes (Fig. 3). The activated catalyst with adsorbed methane was used as the background. Upon elevating the temperature to 300 °C, the intensified negative peaks of 3,016 and 1,304 cm^−1^ represent the stretching vibration and in-plane bending vibration of methyl C–H bond, indicative for the desorption of methane. When achieving 350 °C, the positive peak of 3,003 cm^−1^ arises, corresponding to the methyl stretching vibration. In addition, the positive peak of 1,541 cm^−1^ is indicative for the stretching vibration of the –C=C– fragment. Without any signal assigned to methylene, combining with the gas chromatography-mass spectrometry result, the product was verified as propylene. After running at 400 °C for 2 h, the temperature is decreased to 30 °C, and only negative peak of methane remains, indicating no deposition of heavy products. More details are shown in Fig. S4.

**Fig. 4.**
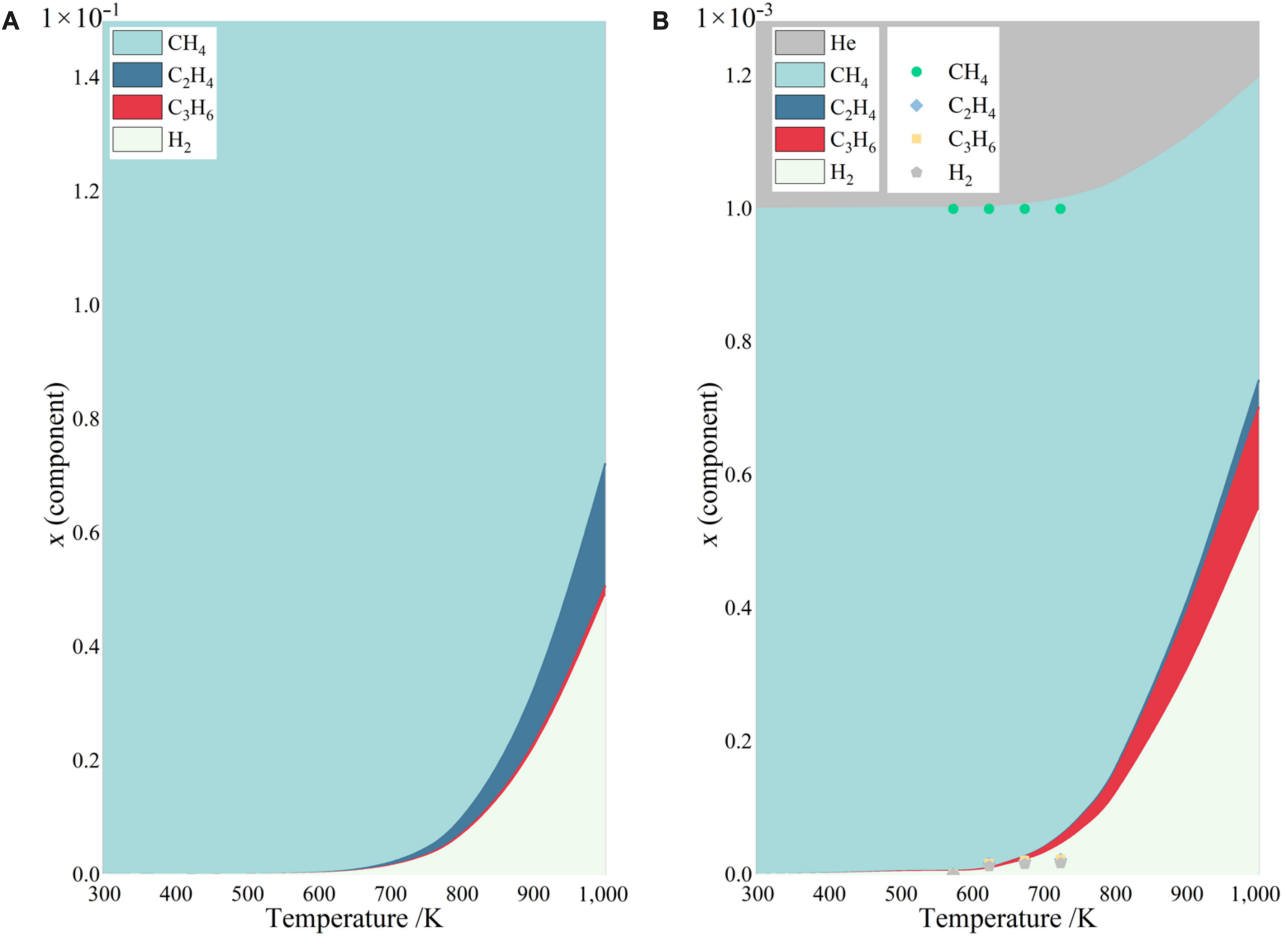
The equilibrium composition diagram for consecutive NOCM processes at (A) 1 atm with 100% methane initial and (B) 1 atm with 0.1% methane and 99.9% He initial. The deep colored points in (B) correspond to the experiment data.

According to the Gibbs–Helmholtz formula, the thermodynamic limit of this work is evaluated. As shown in Fig. 4, considering as coupling consecutive reaction, the NOCM are wildly influenced by the temperature, and it seems that high conversion of methane can be obtained at high temperature, although it should be noted that looping and deposition are not considered. At lower temperature, it shows that the experimentally observed conversion almost approaches its thermodynamic limit (Fig. 4B). Experiments under a temperature of >500 °C was not performed as the catalyst already loses its stability at 500 °C. In addition, Fig. 4 clarified that the presence of inert gas does not affect the equilibrium constant but the equilibrium composition. With the same total pressure, inert gas actually plays a dilution role in the system. According to the Le Chatelier’s principle, with positive stoichiometric coefficient, the NOCM process is favored with higher conversion. In addition, the presence of the inert gas allows for deeper condensation of the system, thus facilitating the production of propylene. Alternatively, the possibility for NOCM proceeding as parallel processes was also considered, while inconsistence was found in kinetic modeling (see Figs. S7 and S8 and Tables S1 to S5 for more details).

**Fig. 5.**
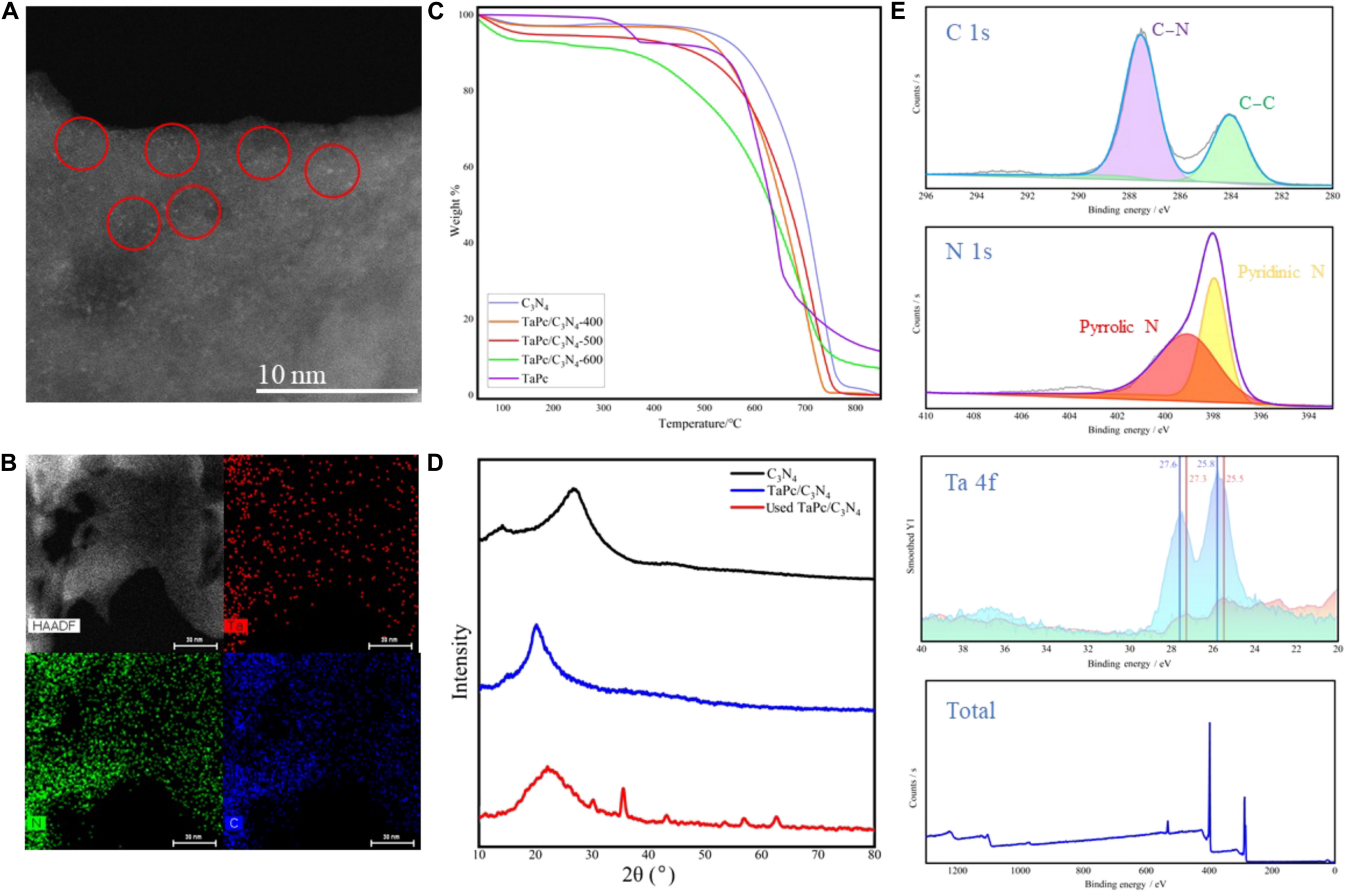
Morphological and structural characterizations of the TaPc/C_3_N_4_. (A) The HADDF-TEM images of TaPc/C_3_N_4_ (0.08 wt%). (B) The corresponding energy-dispersive spectroscopy maps of C, N, and Ta in HADDF-TEM images. (C) Thermogravimetric analyzer spectra of TaPc/C_3_N_4_. (D) X-rays diffraction spectra of TaPc/C_3_N_4_ (0.08 wt%). (E) X-ray photoelectron spectroscopy spectra of TaPc/C_3_N_4_ (0.08 wt%), and Ta 4f for TaPc as reference.

To obtain more structural information of the active catalyst, the high-angle annular dark-field transmission electron microscope (HADDF-TEM) analysis was performed on the TaPc/C_3_N_4_ (0.08 wt%) sample. As shown in Fig. 5A, the bright points in the image indicate atomically dispersed Ta atoms on the surface. Further, the corresponding energy-dispersive spectroscopy maps of the TaPc/C_3_N_4_ heterojunction (Fig. 5B and Fig. S2) reveals the homogeneous distribution of Ta, C, and N across the nanosheet structure, which unambiguously demonstrates that TaPc are uniformly distributed on g-C_3_N_4_ surfaces. In addition, we also tried to perform the extended x-ray absorption fine structure or x-ray absorption near-edge structure analysis on the TaPc/C_3_N_4_ (0.08 wt%) sample; unfortunately, however, no useful information was obtained because of the rather low Ta content.

The thermal stabilities of different components of the catalysts were examined with thermogravimetric analyzer (TGA) analysis (Fig. 5C). The g-C_3_N_4_ material obtained via pyrolysis at 550 °C is thermally stable until 520 °C and completely decomposes at 750 °C. The TaPc complex undergoes a mass loss of 8.2% at 320 °C and is then kept stable until 550 °C; further decomposition occurs at 640 °C, leaving 11.52% ash eventually. The TaPc/C_3_N_4_ samples prepared at 400 and 500 °C have a good thermal stability below 500 °C, but the one prepared at 600 °C starts to lose mass at around 420 °C, probably because the backbone structure of the samples had already been destroyed during the preparation process. Considering the performances of different components of the TaPc/C_3_N_4_ sample under TGA, most likely, the impregnation–pyrolysis (<500°C) sequence retains the Ta–N_4_ structure of TaPc on the g-C_3_N_4_ surface.

The x-rays diffraction patterns show that the typical g-C_3_N_4_ diffraction peak at 27.5° offsets to 21.3° upon stacking the carbon nitride plane to form the TaPc/C_3_N_4_ (0.08 wt%) catalyst [48]. This means that the interplanar spacing increases by inserting TaPc (Fig. 4D). After the catalyst is deactivated, the peak at 21.3° shifts to 22.3° and is less sharp, indicative for a decrease in the interplanar spacing of g-C_3_N_4_. Furthermore, there appear new diffraction peaks in the deactivated catalyst that are identified as C_3_N_4_ (35.6°, 57.0°, and 62.6°) (00-053-0671) [49] and TaN_0.83_ (30.8° and 35.3°) (01-089-4765) [50], respectively. Thus, most likely, the deactivation of the catalyst results from the aggregation of dispersed Ta species to small TaN clusters.

In addition, the electronic morphology of the TaPc/C_3_N_4_ (0.08 wt%) catalyst was probed with x-ray photoelectron spectroscopy analysis. As shown in Fig. 5E, high-resolution C 1s scan shows the evidence of C–C bonding (284.2 eV) and C–N bonding (287.3 eV) and with proportions of 36.3% and 63.7%, respectively; high-resolution N 1s scan shows the pyridinic N (397.9 eV; 54.8%) and pyrrolic N (399.0 eV; 45.2%), respectively. Compared to TaPc, the TaPc/C_3_N_4_ (0.08 wt%) spectrum has a red shift for Ta, which means that the electron density of Ta is increased, indicative for unstacked TaPc species.

**Fig. 6.**
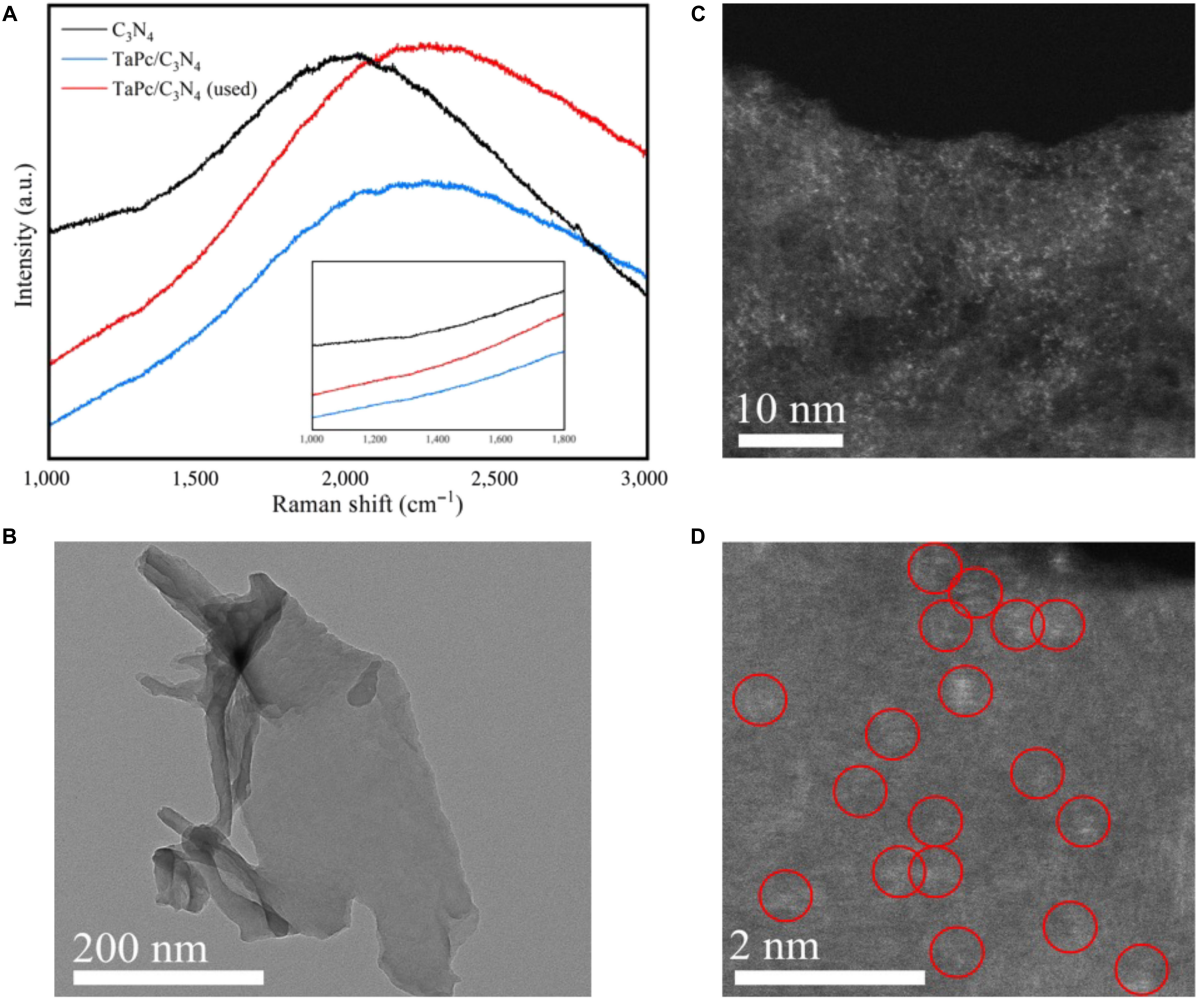
(A) InVia Raman spectra of the inactivation TaPc/C_3_N_4_ (0.08 wt%) catalysts (used after 300 h at 350 °C). (B) The TEM images of used TaPc/C_3_N_4_ (0.08 wt%). (C and D) The HADDF-TEM images of used TaPc/C_3_N_4_ (0.08 wt%) at different resolution ratios. a.u., arbitrary units.

It should be noted that after the catalyst is reactivated, the initial activity and lifetime (Fig. 2B) cannot be recovered, implying the irreversible conversion of the Ta–N_4_ units to the TaN clusters. Further, in the Raman spectrum of the deactivated catalyst, there is no peak at 1,300 and 1,600 cm^−1^ indicative for the D_1_ and G bands of aromatic carbon (Fig. 6A), indicating that coking does not occur. Identically, in the TEM images, the catalyst is still a thin slice with little crimp (Fig. 6B). However, further through the HADDF-TEM images, it is obvious that the metal atoms are gathered with each other (Fig. 6C and D), which bearing out the formation of TaN clusters.

Further, quantum chemical calculations were performed to probe the origins of the excellent performance of the TaPc/C_3_N_4_ catalyst. Considering the preparation procedures and the characteristic results, the macrocyclic structure of TaPc is probably maintained, and a model with TaPc(C_32_N_8_H_16_Ta) supported on C_3_N_4_ (C_90_N_123_H_15_) via π–π stacking was thus built (Fig. 1 and Fig. S10). To perform feasible calculation on such a large model, the semiempirical extended tight-binding computation method [51,52] as developed by Grimme group was used in conjunction with the gau_xtb code [53]. Here, we focused on why ethylene and propylene correspond to the major product.

**Fig. 7.**
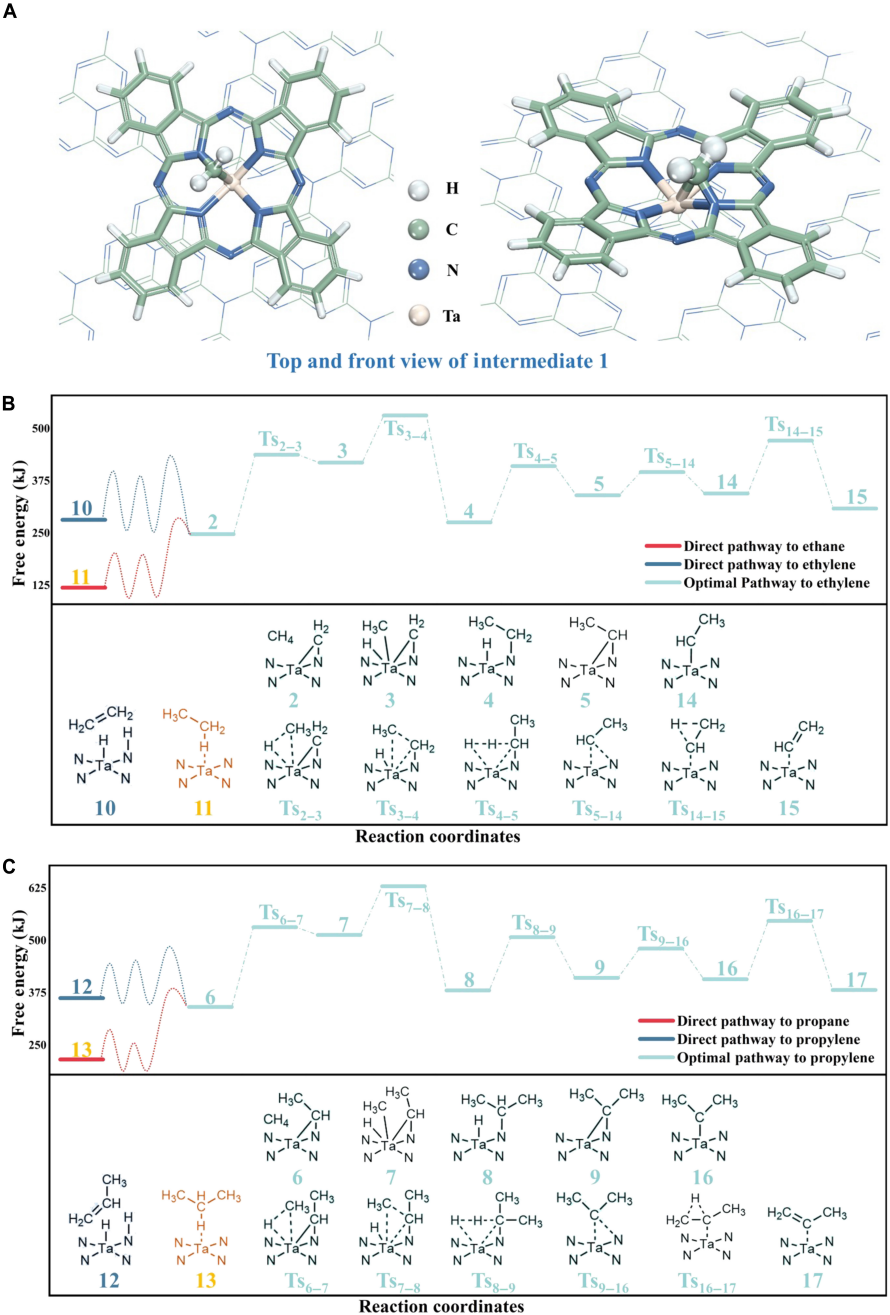
Density functional theory calculation for activating methane of TaPc/C_3_N_4_. (A) The optimized structure of intermediate 1. (B and C) The superior pathway Gibbs energy scheme with each step including transition state. Thermal correction changes from 0 to 300 K.

According to the calculation, the most stable structure for the dehydrogenation of the first CH_4_ molecule correspond to a bridge N–CH_2_–Ta structure (intermediate 1, Fig. 7A). Starting from this intermediate, the conversion of another 2 CH_4_ molecules proceeds similarly. For example, as shown in Fig. 7B, activation of the second CH_4_ occurs via insertion of Ta into the CH_3_–H bond, generating **3**; this is followed by migration of the methyl group from Ta to the CH_2_ unit of the first CH_4_ (**3** → **4**); next, the so-formed ethyl group delivers a hydrogen to the hydride ligand of Ta to extrude molecular hydrogen (**4** → **5**), affording the N–CH(CH_3_)–Ta structure. The conversion of the third CH_4_ precisely resembles the **2** → → **5** sequence, thus proceeding along the **6** → → **9** path. Meanwhile, the formation of the N–CH(CH_3_)–Ta and N–C(CH_3_)_2_–Ta structures may compete with alternative channels. As shown in Fig. 7B and C, via a series of transformation, the C_2_H_4_, C_2_H_6_, C_3_H_6_, and C_3_H_8_ molecules can be released from the reaction system via the sequences **2** → → **10/11** and **6** → → **12/13**, respectively (for intact processes, see Figs. S11 to S13); note that although some of these processes are energetically more favorable as compared to the generation of N–CH(CH_3_)–Ta and N–C(CH_3_)_2_–Ta, all of them are entropically much less favorable as compared to the short paths **2** → → **5** and **6** → → **9**. As a result, most likely, the N–CH(CH_3_)–Ta and N–C(CH_3_)_2_–Ta structures are the major intermediates the system goes through. However, these 2 structures may undergo further isomerization to release neutral olefin molecules: By shifting a hydrogen from the methyl group to the methylene carbon, the intact C_2_H_4_ or C_3_H_6_ is generated (**2** → → **14** and **6** → → **15**). Obviously, such 2 isomerization processes are highly competitive. As to the activation of a fourth CH_4_ molecule, although it is kinetically feasible at the structure **16**, the lack of hydrogen in the bridging carbon prevents the conversion of the fourth methane from a sequential C–C coupling and H_2_ elimination process. Consequently, the propagation of the carbon chain stops at a number 3, and C_3_H_6_ is released as a major product. More details are provided in the Supplementary Materials. Although we tried to calculate as many as possible pathways, there might exist other various routes; after all, the generation of C_2_ and C_3_ involves multiple substrates as well as a series of bond breaking and making processes.

## Conclusion

In summary, a catalyst with single Ta–N_4_ center anchored in phthalocyanine and supported on g-C_3_N_4_ was prepared. Such a catalyst is able to convert methane to ethylene and propylene at a relatively low temperature (350 °C) with the latter as the major product. A TOF value of 0.99 s^−1^ and >300-h one-pass lifetime prove the robustness of the catalyst. The deactivation results from the transformation of the Ta–N_4_ units to TaN clusters during the reaction process. Quantum chemical calculations indicate that the bridge N–CR_2_–Ta (R = H and CH_3_) structures serve as the key intermediates, which either enable the carbon-chain propagation or, alternatively, isomerize to release olefin molecules. Considering the vital role the Ta atom plays in the transformation that it serves as the courier to deliver both carbon and hydrogen, most likely, here, the relativistic effects induced strong Ta–C/H interaction functions once again. Future efforts may focus on how to uniformly load higher content of well-dispersed Ta on a carbon-based material and how to enhance the chemical stability of the Ta–N_4_ structure. Further, the relativistic effects exert similar influence on the gas and condensed phases, which encourages us to continue the gas-phase guided construction of high-performance catalyst. Indeed, gas-phase studies allow us to correlate various structural/electronic features with the performance of the active center, while the major difficulty is still on how these favorable features are replicated in bulk systems. As to the direct conversion of methane to higher hydrocarbons, most likely, a 5d element-centered structure is necessary so as to bind the carbonide intermediates strongly for further propagation of the carbon chain.

## Methods

### The synthesis of g-C_3_N_4_

A mixture of cyanuric acid and melamine was heated to 550 °C with a rate of 5 °C/min in a tube furnace under Ar atmosphere, and the temperature was maintained at 550 °C for 5 h. After cooling to room temperature, faint yellow product of g-C_3_N_4_ was obtained.

### The synthesis of TaPc/C_3_N_4_

A mixture of phthalonitrile, tantalum pentachloride, and *n*-butanol was up to 100 °C, and a certain amount of DBU was next added. The system was stirred under reflux for 6 h in N_2_ atmosphere. The so-prepared reaction solution was mixed with g-C_3_N_4_ at N_2_ atmosphere and stirred for 24 h. After removing the solvent with vacuum evaporation, green solid was obtained. The dried green solid was calcinated in a tube furnace for 5 h to afford TaPc/C_3_N_4_. More technical details are provided in the Supplementary Materials.

### General procedures for methane conversion

In catalytic performance, the catalyst was first activated under reductive atmosphere (10% H_2_/90% He) in a quartz tube furnace. Next, the reactant flow (0.1% CH_4_/99.9% He) was fed in continuously under different temperatures. The product was analyzed using online gas chromatography. The reactivation of the catalysts was performed with calcination under reductive atmosphere (10% H_2_/90% He). For in situ IR analysis, the catalyst was placed in a reaction cell of the spectrometry and subjected to the same condition as that in methane conversion experiment. More technical details are provided in the Supplementary Materials.

### Computational section

The structural optimization and frequency analysis were performed at the GFN1-xTB level of theory using the xTB package (version 6.6.0) [54,55] as interfaced into the Gaussian 09 [56] program using the gau_xtb code [57]. Unscaled vibrational frequencies were used to correct the relative energies for zero-point vibrational energy (ZPVE) contributions. A model of TaPc(C_32_N_8_H_16_Ta) supported on C_3_N_4_ (C_90_N_123_H_15_) via π–π stacking was built. Only the edge atoms of the C_3_N_4_ plane were frozen to avoid any boundary effects. More technical details are provided in the Supplementary Materials.

## Data Availability

Data will be made available on request.

## References

[1] Karakaya C, Kee RJ. Progress in the direct catalytic conversion of methane to fuels and chemicals. Prog Energy Combust Sci. 2016;55:60–97.

[2] Ravi M, Ranocchiari M, van Bokhoven JA. The direct catalytic oxidation of methane to methanol-a critical assessment. Angew Chem Int Ed. 2017;56(52):16464–16483. 10.1002/anie.201702550 28643885

[3] Bottino A, Comite A, Capannelli G, Di Felice R, Pinacci P. Steam reforming of methane in equilibrium membrane reactors for integration in power cycles. Catal Today. 2006;118(1):214–222.

[4] Chen Y, Mu X, Luo X, Shi K, Yang G, Wu T. Catalytic conversion of methane at low temperatures: A critical review. Energ Technol. 2020;8(8): 1900750.

[5] Galvis HMT, de Jong KP. Catalysts for production of lower olefins from synthesis gas: A review. ACS Catal. 2013;3(9):2130–2149.

[6] Annapragada AV, Gulari E. Fe-P-O catalysts for methane utilization – Catalyst development and identification. J Catal. 1990;123(1):130–146.

[7] Dedov AG, Loktev AS, Moiseev II, Aboukais A, Lamonier JF, Filimonov IN. Oxidative coupling of methane catalyzed by rare earth oxides: Unexpected synergistic effect of the oxide mixtures. Appl Catal A Gen. 2003;245(2):209–220.

[8] Schwach P, Pan XL, Bao XH. Direct conversion of methane to value-added chemicals over heterogeneous catalysts: Challenges and prospects. Chem Rev. 2017;117(13):8497–8520. 28475304 10.1021/acs.chemrev.6b00715

[9] Xiao Y, Varma A. Highly selective nonoxidative coupling of methane over Pt-Bi bimetallic catalysts. ACS Catal. 2018;8(4):2735–2740.

[10] Majhi S, Mohanty P, Wang H, Pant KK. Direct conversion of natural gas to higher hydrocarbons: A review. J Energy Chem. 2013;22(4):543–554.

[11] Liu Y, Chen Y, Jiang W, Kong T, Pedro HCC, Gao C, Xiong Y. Highly efficient and selective photocatalytic nonoxidative coupling of methane to ethylene over Pd-Zn synergistic catalytic sites. Research (Wash D C). 2022;2022: 9831340. 36452434 10.34133/2022/9831340 PMC9680520

[12] Guo XG, Fang GZ, Li G, Ma H, Fan HJ, Yu L, Ma C, Wu X, Deng DH, Wei MM, et al. Direct, nonoxidative conversion of methane to ethylene, aromatics, and hydrogen. Science. 2014;344(6184):616–619. 24812398 10.1126/science.1253150

[13] Gerceker D, Motagamwala AH, Rivera-Dones KR, Miller JB, Huber GW, Mavrikakis M, Dumesic JA. Methane conversion to ethylene and aromatics on PtSn catalysts. ACS Catal. 2017;7(3):2088–2100.

[14] Belgued M, Pareja P, Amariglio A, Amariglio H. Conversion of methane into higher hydrocarbons on platinum. Nature. 1991;352(6338):789–790.

[15] Horz G, Lindenmaier K. Kinetics and mechanisms of absorption of carbon by niobium and tantalum in a methane or acetylene stream. J Less-Common Met. 1974;35(1):85–95.

[16] Ranasinghe YA, MacMahon TJ, Freiser BS. Formation of thermodynamically stable dications in the gas phase by thermal ion-molecule reactions: Tantalum^2+^ and zirconium^2+^ with small alkanes. J Phys Chem. 1991;95(20):7721–7726.

[17] Parke LG, Hinton CS, Armentrout PB. Energetics and mechanisms of C−H bond activation by a doubly charged metal ion: Guided ion beam and theoretical studies of Ta^2+^ + CH_4_. J Phys Chem A. 2008;112(42):10469–10480. 18826293 10.1021/jp8052295

[18] Zhu XW, Xu FJ, He Q, Xing Z, Zhang SC, Zhang XR. Detection of intermediates for diatomic TaO^+^ catalyzed gas-phase reaction of methane coupling to ethane and ethylene by ICP-MS/MS. Microchem J. 2021;161: 105762.

[19] Zhou SD, Li JL, Schlangen M, Schwarz H. Differences and commonalities in the gas-phase reactions of closed-Shell metal dioxide clusters MO_2_^+^ (M=V, Nb, and ta) with methane. Chem Eur J. 2016;22(21):7225–7228. 27062433 10.1002/chem.201600498

[20] Zhou SD, Li JL, Schlangen M, Schwarz H. Spin-selective thermal activation of methane by closed-Shell TaO_3_^+^. Angew Chem Int Ed. 2016;55(25):7257–7260. 10.1002/anie.201601965 27159562

[21] Zhou SD, Li JL, Schlangen M, Schwarz H. Efficient room-temperature activation of methane by TaN^+^ under C-N coupling. Angew Chem Int Ed. 2016;55(38):11678–11681. 10.1002/anie.201606259 27510819

[22] Yue L, Wang N, Zhou S, Sun X, Schlangen M, Schwarz H. The electric field as a “smart” ligand in controlling the thermal activation of methane and molecular hydrogen. Angew Chem Int Ed. 2018;57(44):14635–14639. 10.1002/anie.201805718 29888540

[23] Schwarz H. Relativistic effects in gas-phase ion chemistry: An experimentalist’s view. Angew Chem Int Ed. 2003;42(37):4442–4454. 10.1002/anie.200300572 14520739

[24] Pyykko P. Relativistic effects in chemistry: More common than you thought. Annu Rev Phys Chem. 2016;63:45–64. 10.1146/annurev-physchem-032511-143755 22404585

[25] Irikura KK, Beauchamp JL. Methane oligomerization in the gas-phase by 3rd-row transition-metal ions. J Am Chem Soc. 1991;113(7):2769–2770.

[26] Soulivong D, Norsic S, Taoufik M, Coperet C, Thivolle-Cazat J, Chakka S, Basset J-M. Non-oxidative coupling reaction of methane to ethane and hydrogen catalyzed by the silica-supported tantalum hydride: (≡SiO)_2_Ta−H. J Am Chem Soc. 2008;130(15):5044–5045. 18366170 10.1021/ja800863x

[27] Meng D, Chen C, Yi J, Wu Q, Liang J, Huang Y, Cao R. Migration-prevention strategy to fabricate single-atom Fe implanted N-doped porous carbons for efficient oxygen reduction. Research (Wash D C). 2019;2019: 1768595. 31549046 10.34133/2019/1768595 PMC6750073

[28] Wang J, Yu H, Wei Z, Li Q, Xuan W, Wei Y. Additive-mediated selective oxidation of alcohols to esters via synergistic effect using single cation cobalt catalyst stabilized with inorganic ligand. Research (Wash D C). 2020;2020: 3875920. 32025661 10.34133/2020/3875920 PMC6998037

[29] Wu C, Ding S, Liu D, Li D, Chen S, Wang H, Qi Z, Ge B, Song L. A unique Ru-N4-P coordinated structure synergistically waking up the nonmetal P active site for hydrogen production. Research (Wash D C). 2020;2020: 5860712. 33029589 10.34133/2020/5860712 PMC7521024

[30] Han J, Bian J, Sun C. Recent advances in single-atom electrocatalysts for oxygen reduction reaction. Research (Wash D C). 2020;2020: 9512763. 32864623 10.34133/2020/9512763 PMC7443255

[31] Yang L, Xu H, Liu H, Zeng X, Cheng D, Huang Y, Zheng L, Cao R, Cao D. Oxygen-reconstituted active species of single-atom cu catalysts for oxygen reduction reaction. Research (Wash D C). 2020;2020: 7593023. 33094289 10.34133/2020/7593023 PMC7556117

[32] Bi Q, Yuan X, Lu Y, Wang D, Huang J, Si R, Sui M, Huang F. One-step high-temperature-synthesized single-atom platinum catalyst for efficient selective hydrogenation. Research (Wash D C). 2022;2022: 9140841. 10.34133/2020/9140841 PMC7206892 32426729

[33] Cao C, Zhou S, Zuo S, Zhang H, Chen B, Huang J, Wu X, Xu Q, Zhu Q. Si doping-induced electronic structure regulation of single-atom Fe sites for boosted CO_2_ electroreduction at low overpotentials. Research (Wash D C). 2023;6:0079. 36939451 10.34133/research.0079 PMC10017332

[34] Gingl F, Strähle J. Synthese und Struktur von trichloro(phthalocyaninato)tantal(V) / synthesis and structure of trichloro(phthalocyaninato)tantalum(V). Z Naturforsch B: Chem Sci. 1988;43(4):445–448.

[35] Chisaka M, Ishihara A, Uehara N, Matsumoto M, Imai H, Ota K. Nano-TaO*_x_*N*_y_* particles synthesized from oxy-tantalum phthalocyanine: How to prepare precursors to enhance the oxygen reduction reaction activity after ammonia pyrolysis? J Mater Chem A. 2015;3(32):16414–16418.

[36] Chauke VP, Antunes E, Chidawanyika W, Nyokong T. Photocatalytic behaviour of tantalum (V) phthalocyanines in the presence of gold nanoparticles towards the oxidation of cyclohexene. J Mol Catal A Chem. 2011;335(1):121–128.

[37] Zhang GX, Sebastian D, Zhang XL, Wei QL, Lo Vecchio C, Zhang JH, Baglio V, Wang WC, Sun SH, Arico AS, et al. Engineering of a low-cost, highly active, and durable tantalate-graphene hybrid electrocatalyst for oxygen reduction. Adv Energy Mater. 2020;10(24):0075.

[38] Ishihara A, Chisaka M, Ohgi Y, Matsuzawa K, Mitsushima S, Ota K. Synthesis of Nano-TaO*_x_* oxygen reduction reaction catalysts on multi-walled carbon nanotubes connected via a decomposition of oxy-tantalum phthalocyanine. Phys Chem Chem Phys. 2015;17(12):7643–7647. 25728597 10.1039/c5cp00317b

[39] Orti E, Bredas JL, Clarisse C. Electronic-structure of phthalocyanines - Theoretical investigation of the optical-properties of phthalocyanine monomers, dimers, and crystals. J Chem Phys. 1990;92(2):1228–1235.

[40] Nemykin VN, Lukyanets EA. Synthesis of substituted phthalocyanines. ARKIVOC. 2010;2010(1):136–208.

[41] Law KY. Organic photoconductive materials: Recent trends and developments. Chem Rev. 1993;93(1):449–486.

[42] Iwatsu F. Size effects on the alpha-beta transformation of phthalocyanine crystals. J Phys Chem. 1988;92(6):1678–1681.

[43] Ong W-J, Tan L-L, Ng YH, Yong S-T, Chai S-P. Graphitic carbon nitride (g-C_3_N_4_)-based photocatalysts for artificial photosynthesis and environmental remediation: Are we a step closer to achieving sustainability? Chem Rev. 2016;116(12):7159–7329. 27199146 10.1021/acs.chemrev.6b00075

[44] Oh W, Chang VWC, Hu Z, Goei R, Lim T-T. Enhancing the catalytic activity of g-C_3_N_4_ through me doping (me = cu, co and Fe) for selective sulfathiazole degradation via redox-based advanced oxidation process. Chem Eng J. 2017;323:260–269.

[45] Yu XZ, Lai SJ, Xin SS, Chen S, Zhang XL, She XL, Zhan TR, Zhao XL, Yang DJ. Coupling of iron phthalocyanine at carbon defect site via pi-pi stacking for enhanced oxygen reduction reaction. Appl Catal B Environ. 2021;280: 119437.

[46] An S, Zhang G, Wang T, Zhang W, Li K, Song C, Miller JT, Miao S, Wang J, Guo X. High-density ultra-small clusters and single-atom Fe sites embedded in graphitic carbon nitride (g-C_3_N_4_) for highly efficient catalytic advanced oxidation processes. ACS Nano. 2018;12(9):9441–9450. 30183258 10.1021/acsnano.8b04693

[47] Wang X, Chen X, Thomas A, Fu X, Antonietti M. Metal-containing carbon nitride compounds: A new functional organic-metal hybrid material. Adv Mater. 2009;21(16):1609–1612.

[48] Thomas A, Fischer A, Goettmann F, Antonietti M, Mueller J-O, Schloegl R, Carlsson JM. Graphitic carbon nitride materials: Variation of structure and morphology and their use as metal-free catalysts. J Mater Chem. 2008;18(41):4893–4908.

[49] Zhang Z, Guo H, Xu Y, Zhang W, Fan X. Corrosion resistance studies on α-C_3_N_4_ thin films deposited on pure iron by plasma-enhanced chemical vapor deposition. J Mater Sci Lett. 1999;18(9):685–687.

[50] Schönberg N, Overend W, Munthe-Kaas A, Sörensen N. An X-ray study of the tantalum-nitrogen system. Acta Chem Scand. 1954;8(2):199–203.

[51] Pracht P, Caldeweyher E, Ehlert S, Grimme S. A robust non-self-consistent tight-binding quantum chemistry method for large molecules. ChemRxiv. 2019. 10.26434/chemrxiv.8326202.v1.

[52] Grimme S, Bannwarth C, Shushkov P. A robust and accurate tight-binding quantum chemical method for structures, vibrational frequencies, and noncovalent interactions of large molecular systems parametrized for all spd-block elements (Z = 1 – 86). J Chem Theory Comput. 2017;13(5):1989–2009. 28418654 10.1021/acs.jctc.7b00118

[53] Lu T. *gau_xtb:A Gaussian interface for xtb code 1.0.1*; 2020. http://sobereva.com/soft/gau_xtb

[54] Bannwarth C, Ehlert S, Grimme S. GFN2-xTB-An accurate and broadly parametrized self-consistent tight-binding quantum chemical method with multipole electrostatics and density-dependent dispersion contributions. J Chem Theory Comput. 2019; 15(3):1652–1671. 30741547 10.1021/acs.jctc.8b01176

[55] Grimme S, Bannwarth C, Shushkov P. A robust and accurate tight-binding quantum chemical method for structures, vibrational frequencies, and noncovalent interactions of large molecular systems parametrized for all spd-block elements (Z=1-86). J Chem Theory Comput. 2017;13(5): 1989–2009. 28418654 10.1021/acs.jctc.7b00118

[56] Frisch MJ, Trucks GW, Schlegel HB, Scuseria GE, Robb MA, Cheeseman JR, Scalmani G, Barone V, Mennucci B, Petersson GA, et al. Gaussian 09, Revision D.01, Gaussian Inc., Wallingford CT. 2009.

[57] Tian L. gau_xtb: A Gaussian interface for xtb code. http://sobereva.com/soft/gau_xtb

