# Supplementary material for: Direct Conversion of Methane to Propylene
Source: Research (Wash D C). 2023 Sep 8;6:0218. doi: 10.34133/research.0218 (PMC10489882; doi:10.34133/research.0218)
Supplement: Supplementary 1 — Figs. S1 to S15 Tables S1 to S5 [file research.0218.f1.pdf]

Supporting Information

SUPPORTING INFORMATION 1

SYNTHESIS OF CATALYSTS 2

CHEMICALS 2
SYNTHESIS OF TAPC 2
SYNTHESIS OF G-C_3N_4 2
SYNTHESIS OF TAPC/C_3N_4 2

CHARACTERISTIC OF CATALYSIS 3

TANTALUM PHTHALOCYANINE CONFIRMATION 3
PRODUCT CONFIRMATION 5

EXPERIMENTAL DETAILS 6

GENERAL DETAILS OF EXPERIMENTAL .... ERROR! BOOKMARK NOT DEFINED.
METHOD ABOUT CALCULATING THE THERMODYNAMIC LIMIT 8
CATALYTIC PERFORMANCE DATA 13

COMPUTATIONAL DETAILS 14

REACTION COORDINATES DATA 16
THE STRUCTURES OF REACTION INTERMEDIATE 19

REFERENCE 25

Synthesis of catalysts

Chemicals

Methanol [CH_4O, Sinopharm, CAS#: 67-56-1, purity: AR, ≥99.5%],

Ethanol [C_2H_6O, Sinopharm, CAS#: 64-17-5, purity: AR, ≥99.7%],

n-butanol [$C_4H_{10}O$, Sinopharm, CAS#: 71-36-3, purity: GR, ≥99.5%],

phthalonitrile [$C_8H_4N_2$, Aladdin, CAS#: 91-15-6, purity: 98%],

1,8-Diazabicyclo [5.4.0] undec-7-ene (DBU) [$C_9H_{16}N_2$, Aladdin, CAS#: 6674-22-2, purity: 99%],

tantalum pentachloride n-butanol solution[Aladdin, purity: 200g TaCl5/L solution],

cyanuric acid [$C_3H_3N_3O_3$, Aladdin, CAS#: 108-80-5, purity: 98%],

melamine [$C_3H_3N_6$, Aladdin, CAS#: 108-78-1, purity: 99%].

Synthesis of TaPc

A mixture of n-butanol (20 mL), phthalonitrile (0.4312 g, 3.365 mmol), and tantalum pentachloride n-butanol solution (1.34 mL, 0.748 mmol $TaCl_5$) was mixed in a Schlenk glass tube, which was sealed under N_2 atmosphere and heated. When the temperature reached 100 °C, DBU (24 drops) was added. The system is refluxed and stirred at boiling point for 6 h. Then the solvent vaporized a half though vacuum distillation. After cooling to room temperature, anti-solvent methanol (10 mL) was added and the purple-solid product was obtained. With methanol washing and drying, TaPc was obtained.

Synthesis of g-C_3N_4

A mixture of cyanuric acid (5 g, 38.7 mmol) and melamine (5 g, 39.6 mmol) was heated to 550°C at the rate of 5°C/min in a tube furnace and Ar atmosphere, and was maintained at 550°C for 5 h. after it cooled to room temperature at the rate of 5°C/min, faint yellow product of g-C_3N_4 was obtained.

Synthesis of TaPc/C_3N_4

A mixture of 1-butanol (20 mL), phthalonitrile (0.4312 g, 3.365 mmol), and tantalum pentachloride n-butanol solution (1.34 mL, 0.748 mmol $TaCl_5$) was mixed in a Schlenk glass tube, which was sealed under N_2 atmosphere and heated. When the temperature reached 100°C, DBU (24 drops) was added. The system

is refluxed and stirred at boiling point for 6 h. Finally, 20.3237 g reaction solution was obtained. A mixture of n-butanol (15 mL), reaction solution (0.1758 g) and g-C_3N_4 (0.2 g) was mixed in another Schlenk glass tube, which was impregnate under N_2 atmosphere for 24 h. After impregnation, the solvent was evaporated to dryness, and the green solid was obtained. Then the green solid was placed into a tube furnace and heated to 400°C/500°C/600°C at the rate of 5°C/min under Ar atmosphere for 5 h. After cooled down, the TaPc/C_3N_4 was obtained.

Characteristic of catalysis

Tantalum phthalocyanine confirmation

The structure of TaPc is confirmed by MALDI-MS method. TaPc – 692; TaOPc – 711

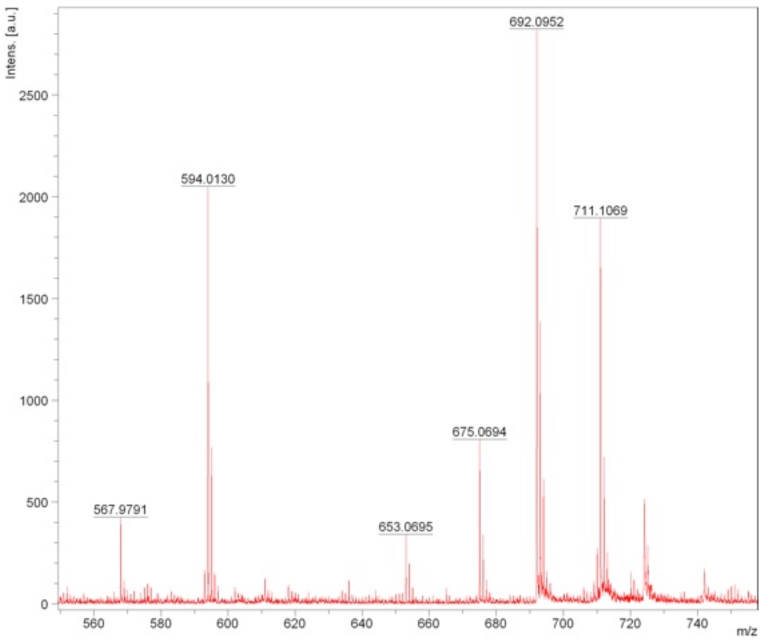

Figure S1 MALDI mass spectra of TaPc

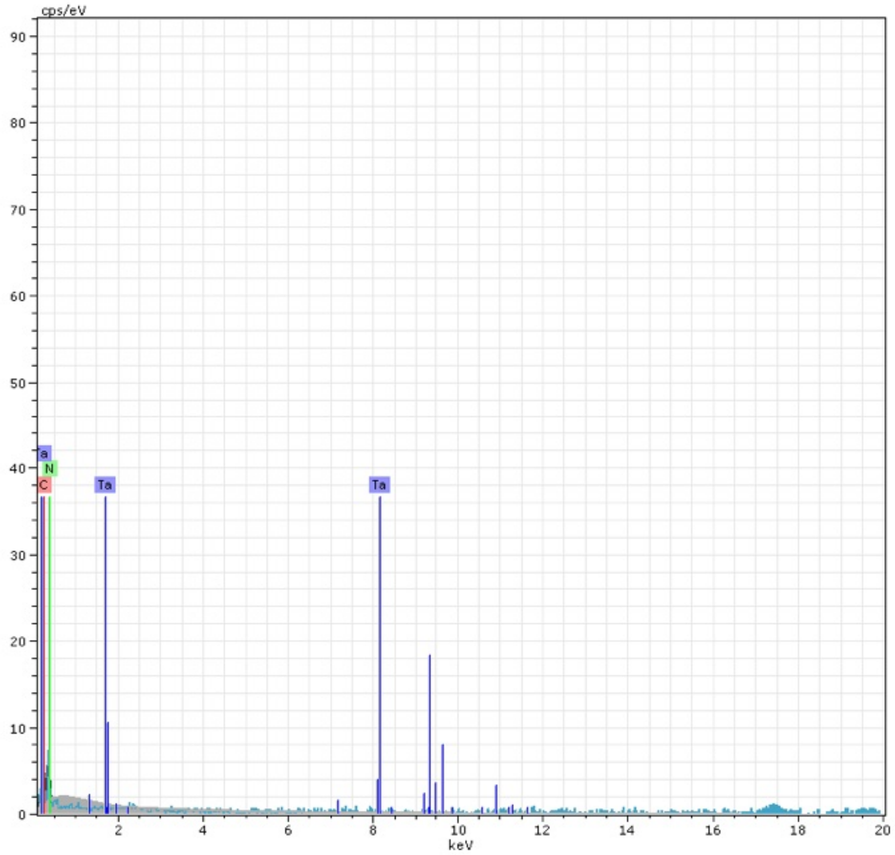

Figure S2 The Eds quantified chart

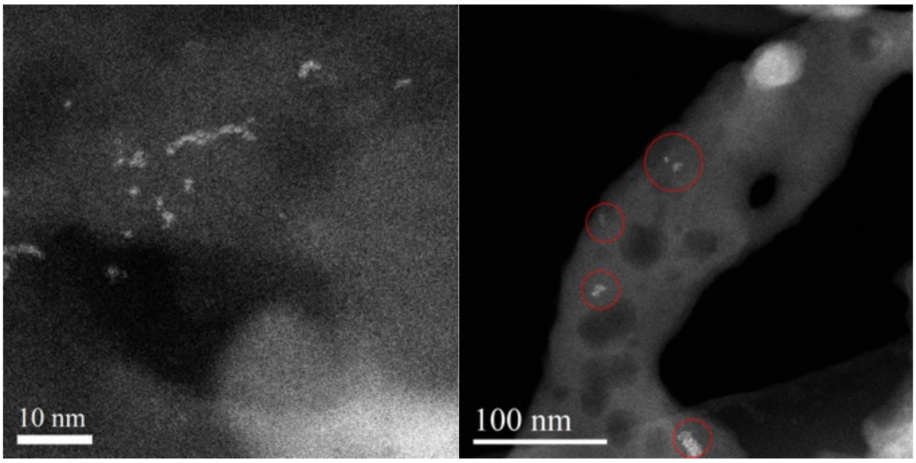

Figure S3 The structural characterizations of the TaPc/C_3N_4 of high loads sample (7 wt.%)

Product confirmation

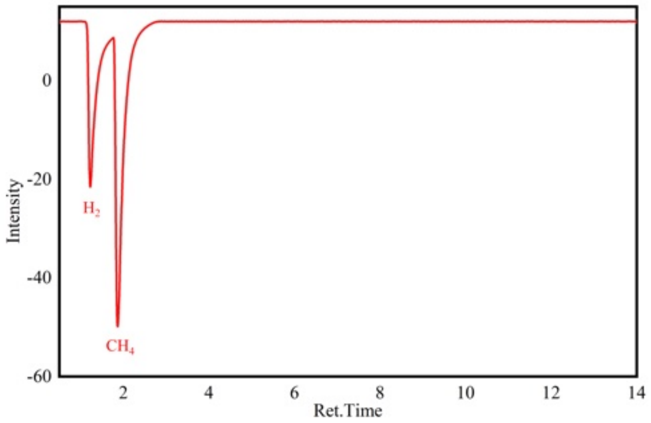

Figure S4 online GC spectra of H_2, detector: TCD; carrier gas: Argon; column: 5A MS packed column.

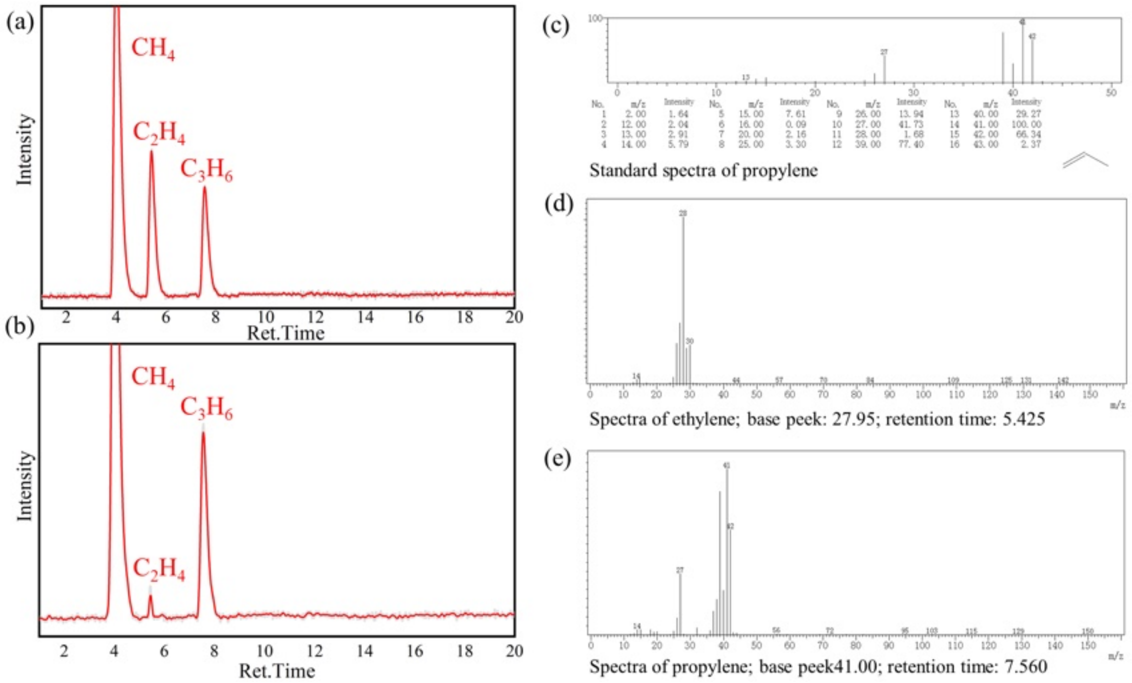

Figure S5 (a) GC-MS spectra of product at 20 min.; (b) GC-MS spectra of product after 2 hours; (c) Standard spectra of propylene; (d) MS spectra of (a) in RT 5.425 min.; (e) MS spectra of (a) in RT 7.560 min.

The retention time of the main peak are 4.065, 5.425 and 7.560 min. The base peak of the mean product at 7.560 min. is 41 m/z, indicating the fragment of $C_3H_7^+$, combining to the molecule of propylene, which is also coincide to standard composition gas peek.

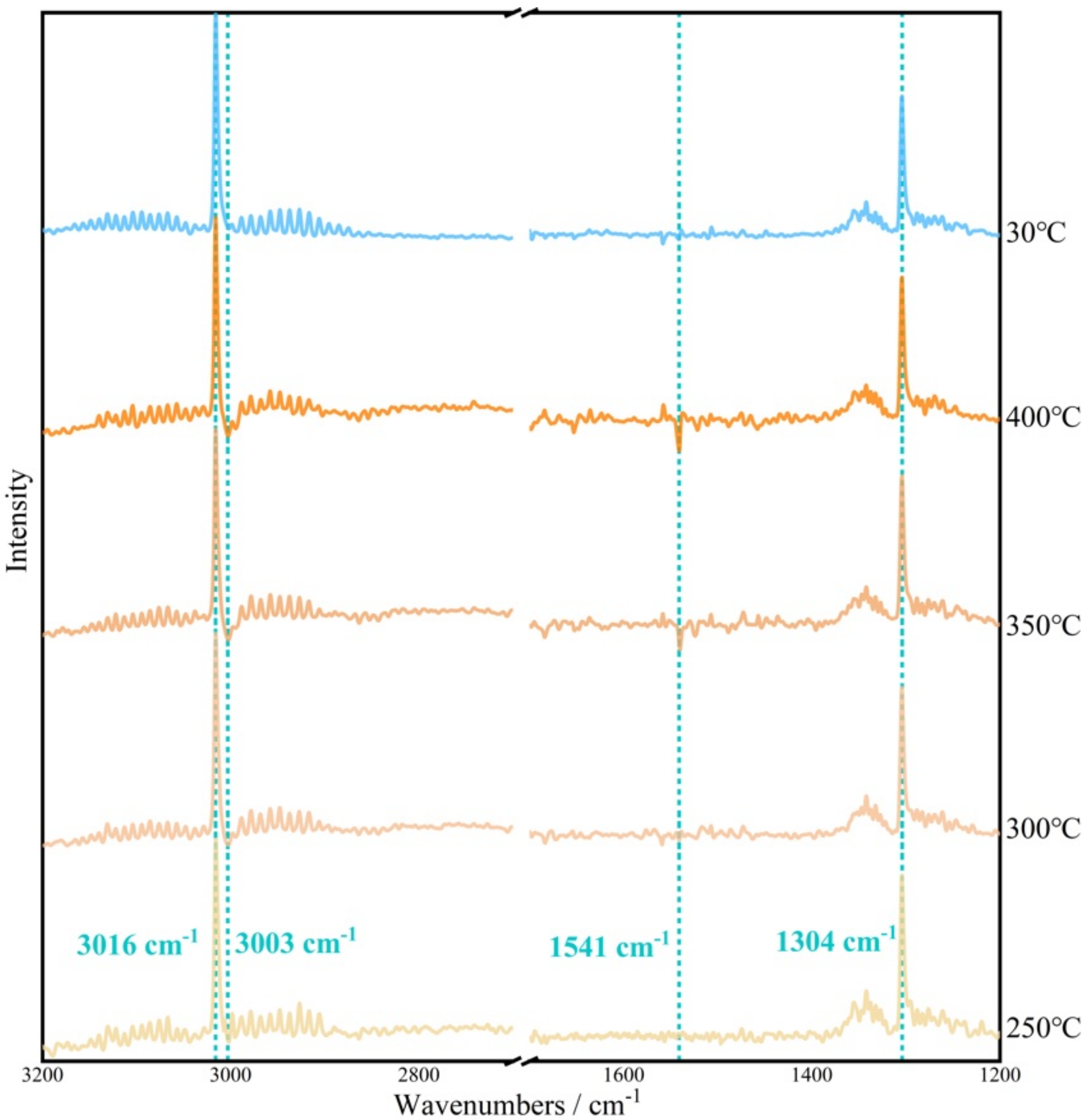

Figure S6 The enlarged in situ DRIFTS with temperature gradient of TaPc/C_3N_4 (0.08wt%)

Experimental details

Instruments: MALDI-MS (BRUKER ImagePrep); online GC (quartz tube furnace connected with agilent 7890A with online detect modified), column (HT-PLOT Q); GC-MS (shimadzu GCMS-QP2010 Ultra), column (PoraBOND Q with PT modified); in-situ DRIFTS (Thermofisher Nicolet iS50) with MCT detector; HADDF-TEM (FEI Titan ChemiSTEM); TGA (Perkin-Elmer Pyris 1 TGA); XRD (Bruker APEXII); XPS (Thermofisher Escalab 250Xi); InVia Raman (Renishaw inVia-reflex)

Catalytic performance: the catalyst was first under a 3 mL/s flow (10% H_2 / 90% He, control by mass flowmeter, similarly hereinafter) in quartz tube furnace connected with online gas chromatography for 12

h in 400°C, 500°C or 600 °C (decided with preparation temperature) to activization. Then, a 10 mL/s He flow purging for 2h in 200 °C, after H_2 undetected, catalytic performance could be start. Then detected gas composition after reacting with a 1.5 mL/s flow (0.1% CH_4 / 99.9% He) at different temperature (300°C, 350°C, 400 °C and 450 °C) for 4 h, respectively.

Proper control experiments: Same to the catalytic performance experiments, but use empty pipe, only g-C_3N_4 and pure Tantalum phthalocyanine, respectively.

Lifetime performance for TaPc/C_3N_4 (0.08wt%): the catalyst was first under a 3 mL/s flow (10% H_2 / 90% He) in quartz tube furnace connected with online gas chromatography for 12 h in 500°C to activization. Then, a 10 mL/s He flow purging for 2h in 200 °C, after H_2 undetected, lifetime performance could be start. Then detected gas composition after reacting with a 1.5 mL/s flow (0.1% CH_4 / 99.9% He) at 350°C, and 450 °C until the methane conversion is below 1%. Then reactivation it under a 3 mL/s flow (10% H_2 / 90% He) in quartz tube furnace connected with online gas chromatography for 12 h in 500°C, re-purge it, and test again.

The turnover frequency (TOF) was calculated as follows:

$$\mathrm{TOF}=\frac{\text{Reacted molecules}}{\text{Metal atoms}\times\text{time}}=\frac{M_{\mathrm{Ta}}\times Conversion_{\mathrm{CH_4}}\times mflow_{\mathrm{CH_4}}}{M_{\mathrm{CH_4}}\times m_{cat.}\times wt_{\mathrm{Ta}}}$$

Where M_{Ta} is the molar mass of Ta; $Conversion_{\mathrm{CH_4}}$ is the CH_4 conversion; $mflow_{\mathrm{CH_4}}$ is the mass flow of CH_4 (g/s); $M_{\mathrm{CH_4}}$ is the molar mass of CH_4; $m_{cat.}$ is the mass of TaPc/C_3N_4 catalyst; wt_{Ta} is Ta wt% loading.

Product confirmation for TaPc/C_3N_4 (0.08wt%): the catalyst was put in to in situ reaction cell and under the same condition with catalytic performance test. Then collecting IR Spectrum in each temperature.

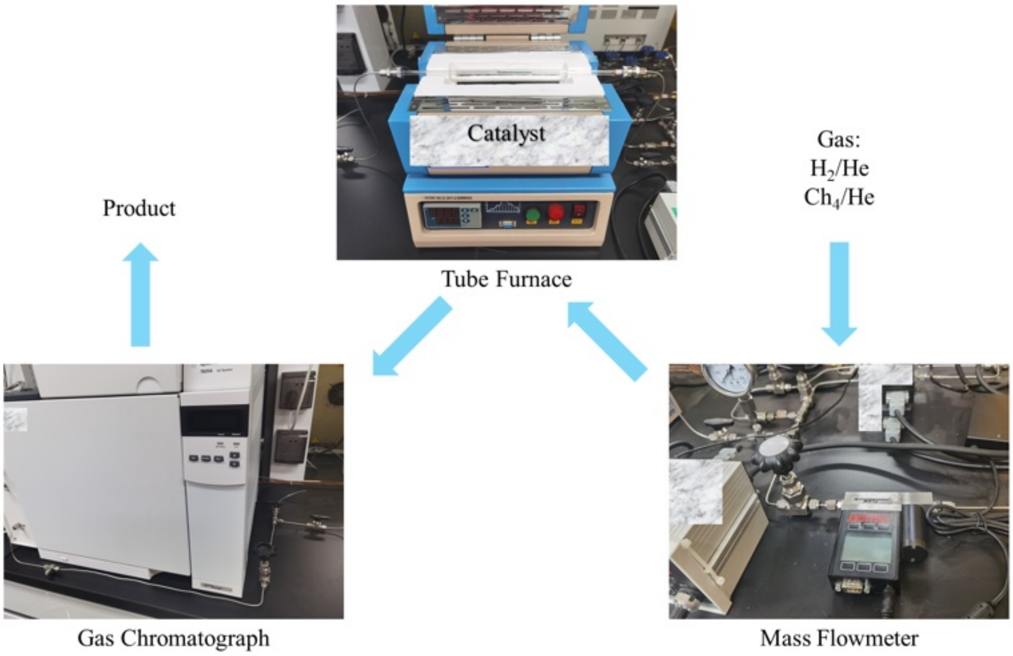

Figure S7 Schematic diagram of catalytic performance experiments.

The calculation of the thermodynamic limit

Here is the method about calculating the thermodynamic limit:

Heat capacity C_p can be written as the function of temperature:

$$C_p = a + bT + cT^2 + \cdots \tag{1}$$

Whereas *a, b, c* are constants.

The difference in isobaric heat capacity between the product and the reactant is:

$$\Delta C_p = \Delta a + \Delta bT + \Delta cT^2 + \cdots \tag{2}$$

Thus

$$\begin{aligned}\Delta H &= \int \Delta C_p \mathrm{d}T + \Delta H_0 \\ &= \Delta H_0 + \int (\Delta a + \Delta bT + \Delta cT^2 + \cdots)\mathrm{d}T \\ &= \Delta H_0 + \Delta aT + \frac{1}{2}\Delta bT^2 + \frac{1}{3}\Delta cT^3 + \cdots\end{aligned} \tag{3}$$

Whereas H_0 is an integration constant, substituting to Gibbs-Helmholtz formula:

$$\frac{\partial\left(\frac{\Delta G}{T}\right)}{\partial T} = \frac{-\Delta H}{T^2} = \frac{-\Delta H_0 - \Delta a T - \frac{1}{2}\Delta b T^2 - \frac{1}{3}\Delta c T^3 - \cdots}{T^2} \tag{4}$$

Transfer and integral,

$$\left(\frac{\Delta G}{T}\right) = \frac{\Delta H_0}{T} - \Delta a \ln T - \frac{1}{2}\Delta b T - \frac{1}{6}\Delta c T^2 + \cdots + I \tag{5}$$

Also shown as

$$\Delta G = \Delta H_0 - \Delta a T \ln T - \frac{1}{2}\Delta b T^2 - \frac{1}{6}\Delta c T^3 + \cdots + IT \tag{6}$$

Whereas I is another integration constant.

The relationship between Gibbs free energy and equilibrium constant:

$$\Delta_r G_m^{\ominus}(T) = -RT \ln K_f^{\ominus} \tag{7}$$

According to the relationship between C_p and T, through equation 3, using the $\Delta_f H$ from a certain temperature can get integration constant H_0. Through equation 6, the other integration constant I can be calculated, which means that the $\Delta_r G_m(T)$ in any temperature can be calculated. With equation 7, the equilibrium constant $K_f^{\ominus}$ can be obtained.

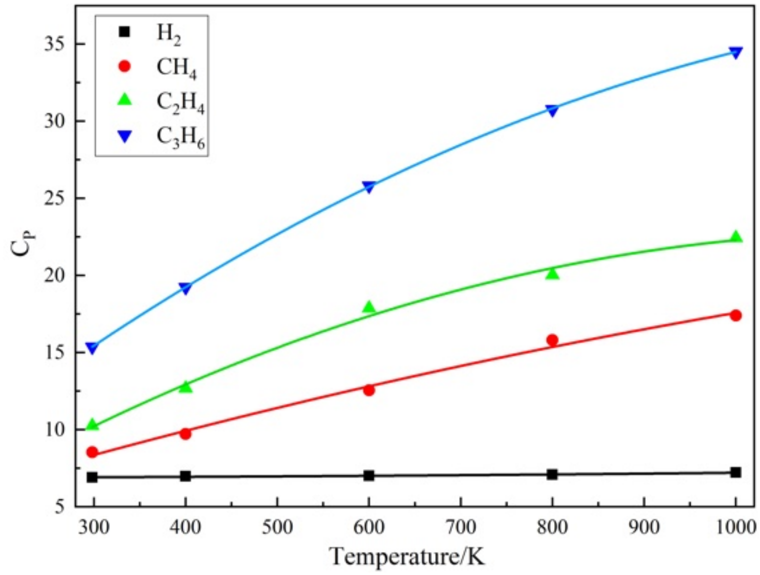

Figure S8 The relationship between isobaric heat capacity and temperature.

For this work, the C_p at different temperatures, $\Delta_f H_{298K}$ and $\Delta_f G_{298K}$ of CH_4, C_2H_4, C_3H_6 and H_2

were obtained from Lange's Handbook of Chemistry[5], and the relationship between C_p and T is shown in Figure S8. Figure S8 was obtained by fitting equation 1, and the values for *a, b* and *c* are shown in Table S1.

Table S1 The fitting values for *a, b* and *c*

Spaces	T<br>K	a<br>Cal·moL^{-1}·K^{-1}	b<br>Cal·moL^{-1}·K^{-1}	c<br>Cal·moL^{-1}·K^{-1}
H_2	298.15	6.87E+00	7.39E-05	2.65E-07
CH_4	298.15	3.16E+00	1.86E-02	-4.18E-06
C_2H_4	298.15	1.47E-01	3.85E-02	-1.64E-05
C_3H_6	298.15	1.95E+00	5.04E-02	-1.78E-05

In this work, the NOCM reaction can be written as coupling consecutive reaction:

	$2CH_4$	$\rightleftharpoons$	C_2H_4	$2H_2$	He
initial	1		0	0	999
reaction	-2x		x	2x	999
equilibrium	$1-2x-y$		$x-y$	$2x+y$	999

	CH_4	C_2H_4	$\rightleftharpoons$	C_3H_6	H_2	He
initial	1	0		0	0	999
reaction	-y	-y		y	y	999
equilibrium	$1-2x-y$	$x-y$		y	$2x+y$	999

where x and y represent the molar fractions of ethylene and propylene, respectively. With the value of *a, b* and *c*, it's easy to get Δa, Δb and Δc; further, thought equation 3 and 6 , H_0 and I can be obtained. All these data are shown in Table S2.

Table S2 The values of equilibrium parameters for consecutive processes

equilibrium	Δa<br>Cal·mol^{-1}·K^{-1}	Δb<br>Cal·mol^{-1}·K^{-1}	Δc<br>Cal·mol^{-1}·K^{-1}	ΔH_0<br>kcal·moL^{-1}	I<br>Cal·mol^{-1}·K^{-1}
$2CH_4 \rightleftharpoons C_2H_4 + 2H_2$	7.57E+00	1.47E-03	-7.48E-06	46.02	25.08
$CH_4 + C_2H_4 \rightleftharpoons C_3H_6 + H_2$	5.51E+00	-6.69E-03	3.01E-06	8.90	37.02

Next, with equation 7, the equilibrium constant in different temperature can be calculated:

Table S3 The equilibrium constant in different temperature of consecutive reaction

T K	equilibrium constant K_f $2CH_4 \rightleftharpoons C_2H_4 + 2H_2$	$CH_4 + C_2H_4 \rightleftharpoons C_3H_6 + H_2$
298.15	1.6513E-30	1.0854E-08
400	1.9744E-21	9.6496E-07
500	4.8746E-16	1.4551E-05
600	2.1389E-12	9.3329E-05
650	5.5440E-11	1.9328E-04
700	9.1613E-10	3.6317E-04
750	1.0539E-08	6.3085E-04
800	9.0184E-08	1.0275E-03
900	3.2918E-06	2.3412E-03

For another possibility, if the reaction were considered as parallel processes:

	$3CH_4$	$\rightleftharpoons$	C_3H_6	$3H_2$	He
initial	1		0	0	999
reaction	$-3y$		b	$3y$	999
equilibrium	$1 - 2x - 3y$		b	$2x + 3y$	999

	$2CH_4$	$\rightleftharpoons$	C_2H_4	$2H_2$	He
initial	1		0	0	999
reaction	$-2x$		a	$2x$	999
equilibrium	$1 - 2x - 3y$		a	$2x + 3y$	999

where x and y represent the molar fractions of ethylene and propylene, respectively. According to the Le Chatelier's principle, reaction 1 is prone to generate ethylene and hydrogen, and reaction 2 is insensitive with pressure. For the consecutive coupling processes, reaction 2 tends to consume ethylene in order to make reaction 1 advantageous. The final product composition is determined by the equilibrium constant of these to reactions. The high value of equilibrium constant thus leads to dominating production of propylene.

Similar to the coupling consecutive reaction, the data are shown below:

Table S4 The values of equilibrium parameters for parallel reaction

equilibrium	Δa Cal·moL^{-1}·K^{-1}	Δb Cal·moL^{-1}·K^{-1}	Δc Cal·moL^{-1}·K^{-1}	ΔH_0 Kcal·moL^{-1}	I Cal·moL^{-1}·K^{-1}
$2CH_4 \rightleftharpoons C_2H_4 + 2H_2$	7.57E+00	1.47E-03	-7.48E-06	46.02	25.08

$3CH_4 \rightleftharpoons C_3H_6 + 3H_2$	1.31E+01	-5.22E-03	-4.47E-06	54.92	62.09

Table S5 The equilibrium constant in different temperature of parallel reaction

T	equilibrium constant K_f	
K	$2CH_4 \rightleftharpoons C_2H_4 + 2H_2$	$3CH_4 \rightleftharpoons C_3H_6 + 3H_2$
298.15	1.6513E-30	1.7924E-38
400	1.9744E-21	1.9052E-27
500	4.8746E-16	7.0929E-21
600	2.1389E-12	1.9962E-16
650	5.5440E-11	1.0715E-14
700	9.1613E-10	3.3272E-13
750	1.0539E-08	6.6488E-12
800	9.0184E-08	9.2660E-11
900	3.2918E-06	7.7068E-09

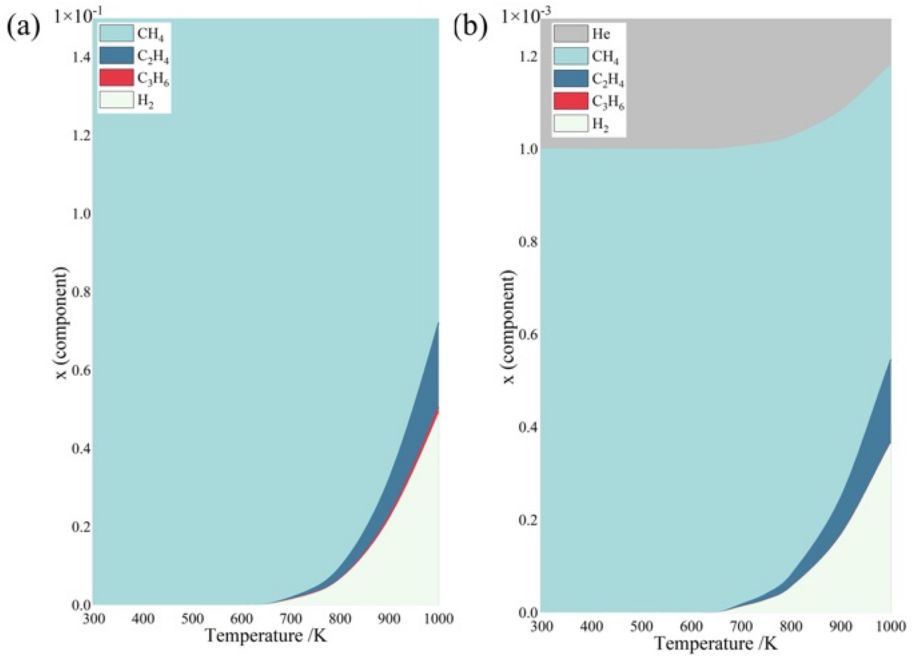

Figure S9 The equilibrium composition diagram of NOCM parallel reaction in (a) 1 atm with 100% methane initial; (b) 1 atm with 0.1% methane and 99.9% He initial.

Catalytic performance data

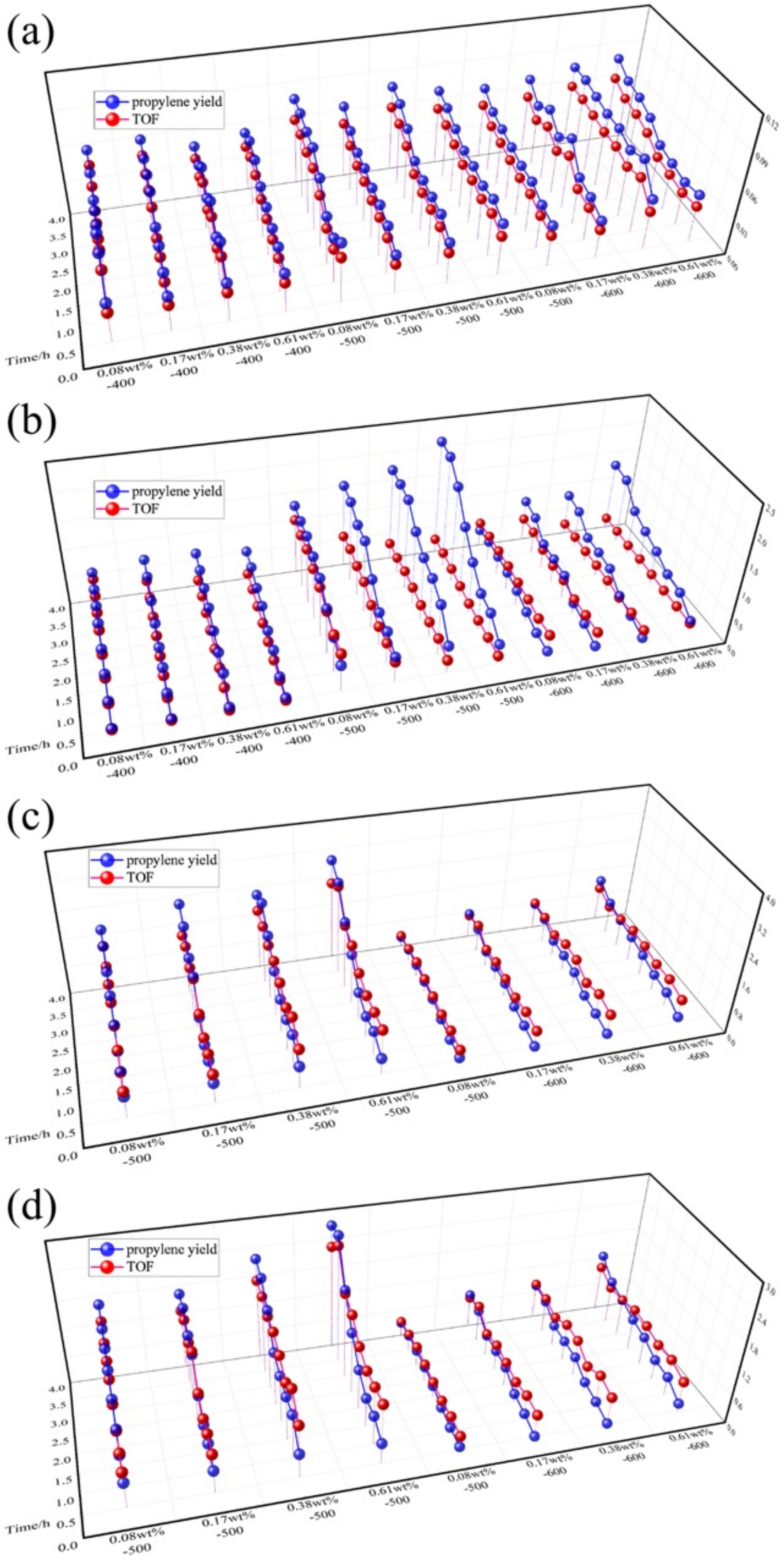

Figure S10 The relationship of temperature, metal contains (Ta wt%), propylene yield and TOF of $TaPc/C_3N_4$ catalysts after 4h, (a) at 300°C; (b) at 350°C; (c) at 400°C; (d) at 450°C.

Computational details

The structural optimization and frequency analysis were performed at the GFN1-xTB level of the xTB package (version 6.6.0) [1-2] as interfaced into the Gaussian 09 [3] program using the gau_xtb code. [4] Stationary points were optimized without symmetry constraint, and their nature was confirmed by vibrational frequency analysis. Unscaled vibrational frequencies were used to correct the relative energies for zero-point vibrational energy (ZPVE) contributions. Considering the preparation procedures and the characteristic results, the macrocyclic structure of TaPc is probably maintained, and a model with TaPc($C_{32}N_8H_{16}Ta$) supported on C_3N_4 ($C_{90}N_{123}H_{15}$) via π-π stacking was thus built. Only at the edge of C_3N_4 atoms were freeze to simulation the slightly rolling surface.

For electronic self-consistent calculations, geometry optimizations and frequency calculations, the convergence tolerance is set as follows: integral cutoff = 0.20E + 02, Broyden damping = 0.40 and the accuracy = 1.0.

The free energy changes for different adsorptions were determined as follows:

$$G_{Ts} = E_{Ts} + \Delta E_{\mathrm{ZPE}} + \Delta H_{0\rightarrow 300\mathrm{K}} - T\Delta S$$

$$G_I = E_I + \Delta E_{\mathrm{ZPE}} + \Delta H_{0\rightarrow 300\mathrm{K}} - T\Delta S$$

$$\Delta \mathrm{G} = \mathrm{G}_{\mathrm{Ts}} - G_I$$

Here, E_{Ts} is the electronic energy for transition states, G_{Ts} is the Gibbs energy for transition states, E_I is the electronic energy of intermediate, G_I is the Gibbs energy of intermediate, while ΔE_{ZPE}, $\Delta H_{0\rightarrow 300\mathrm{K}}$, and ΔS are zero-point energy change, thermal correction change from 0 to 300 K, and entropy change, which were also obtained through the Gaussian 09 program using the gau_xtb code.

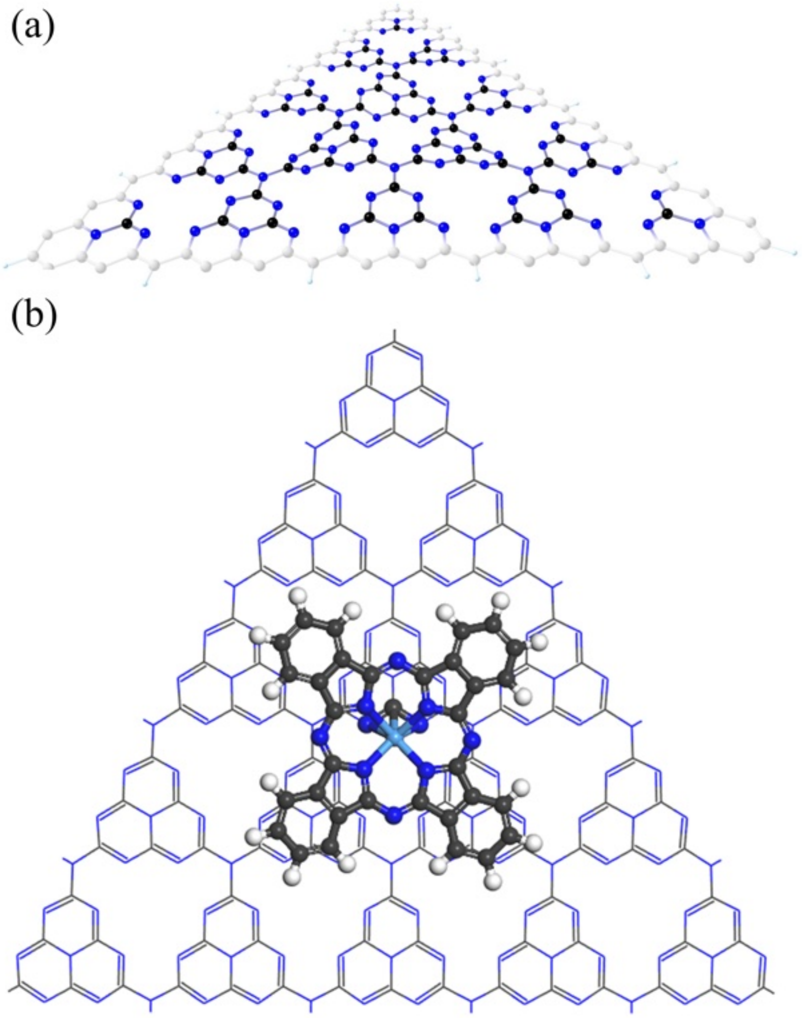

Figure S11 (a) The C_3N_4 model, Gray atoms were freeze; (b) The optimized structure of TaPc/C_3N_4. Blue: N, black: C, cyan: H.

Reaction coordinates data

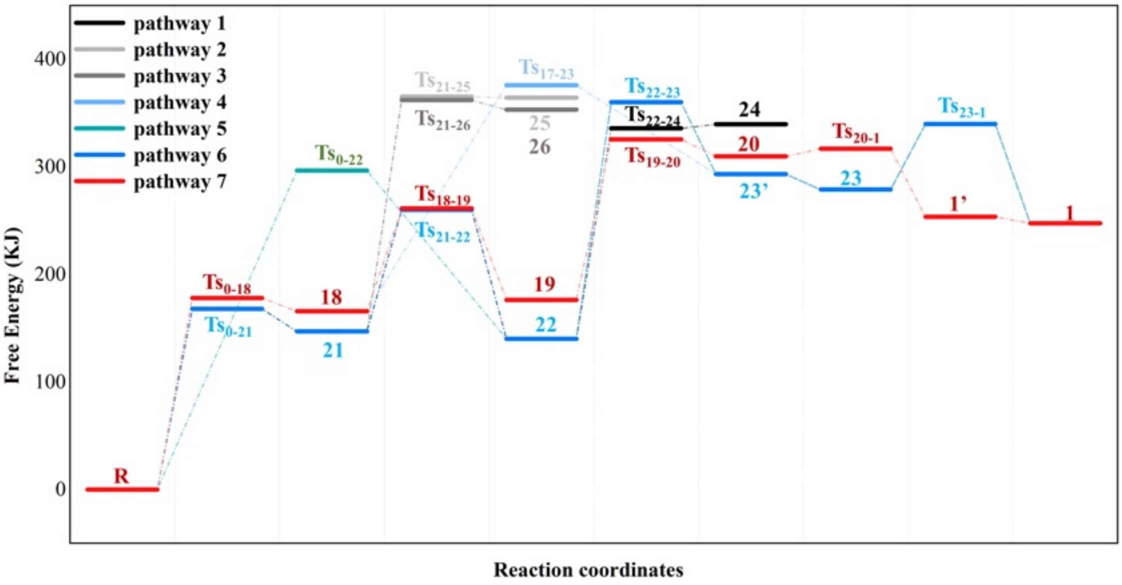

Figure S12 Gibbs energy scheme for first methane activating with each step including transition state.

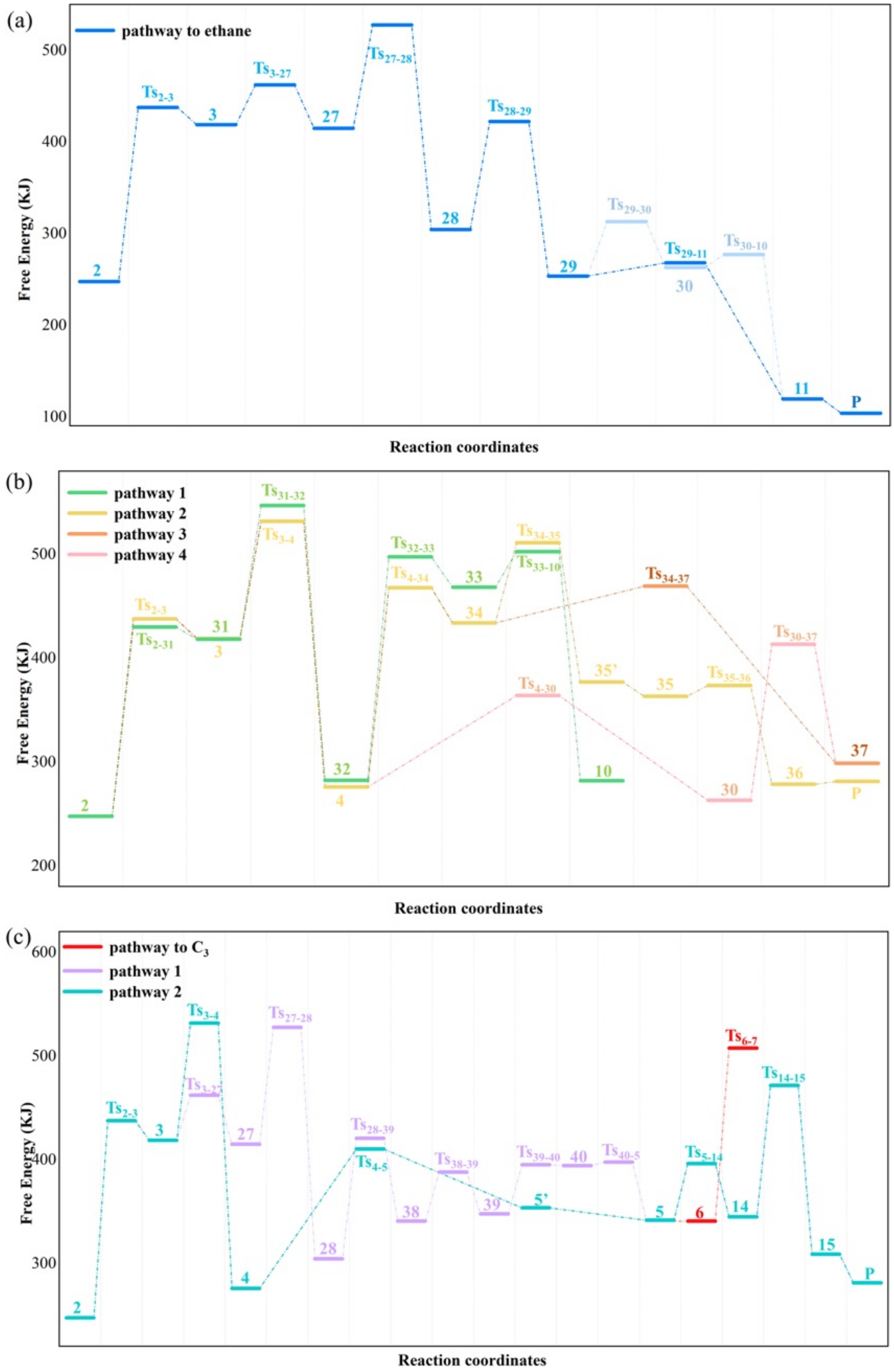

Figure S13 Gibbs energy scheme for second methane activating with each step including transition state. (a) Direct pathway to ethane; (b) Direct pathway to ethylene; (c) Pathway to ethylene by bridge N-CH(CH_3)-Ta.

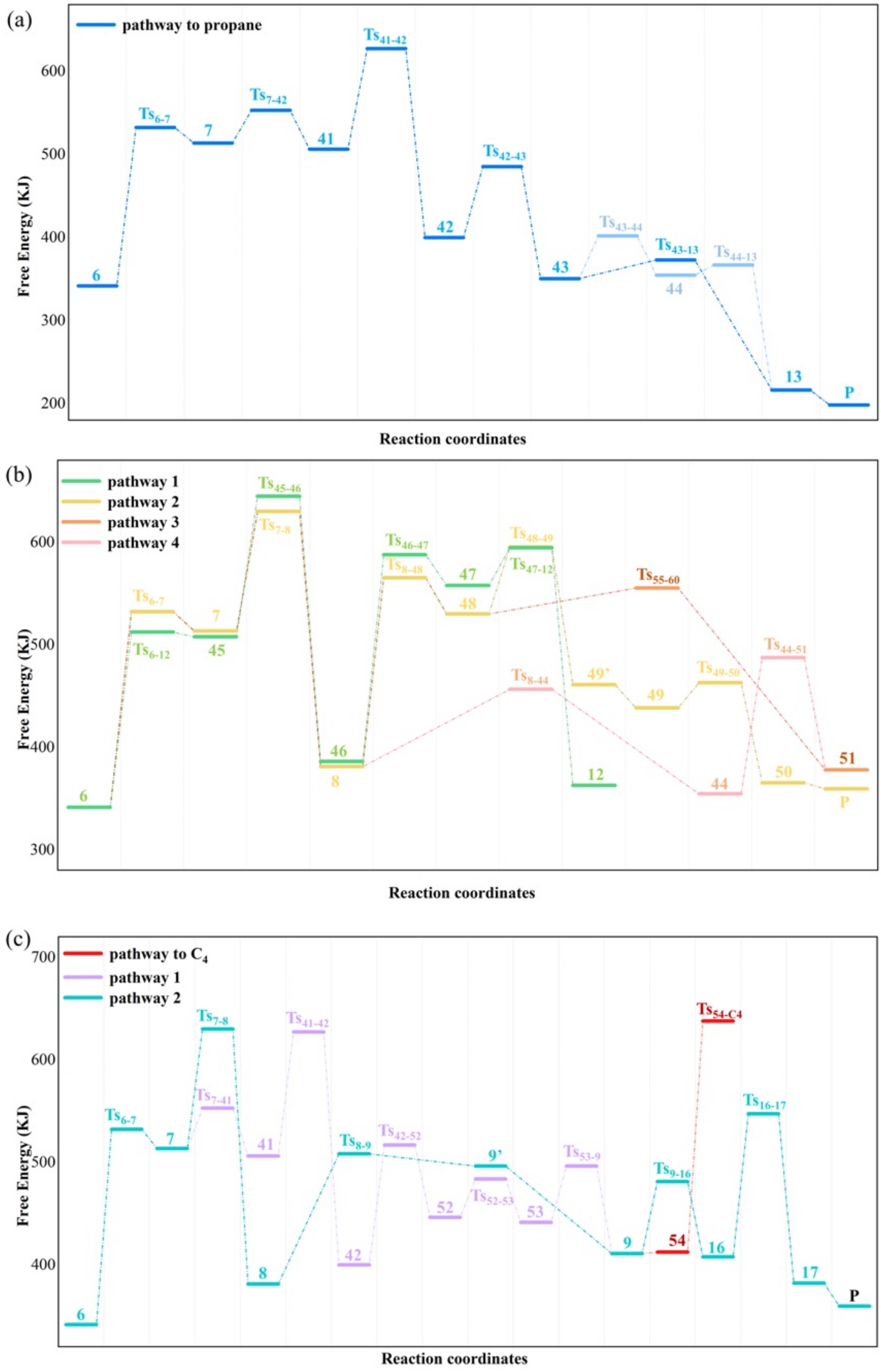

Figure S14 Gibbs energy scheme for second methane activating with each step including transition state. (a) Direct pathway to propane; (b) Direct pathway to propylene; (c) Pathway to propylene by bridge N-C(CH_3)$_2$-Ta.

The structures of reaction intermediate

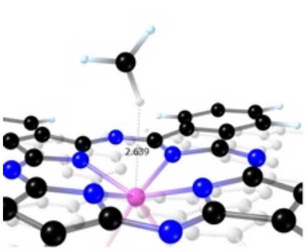

R

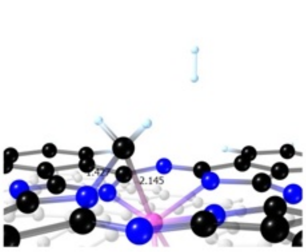

1’

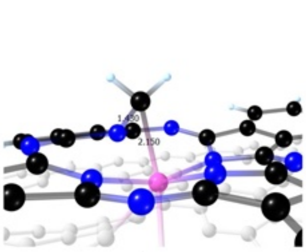

1

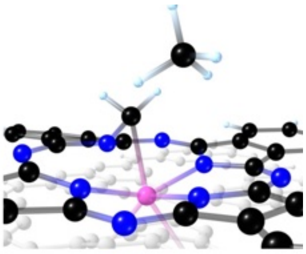

2

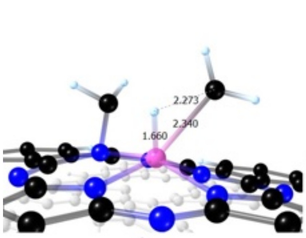

3

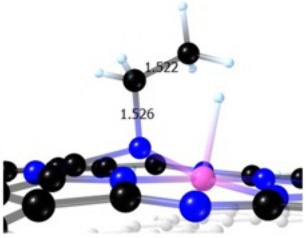

4

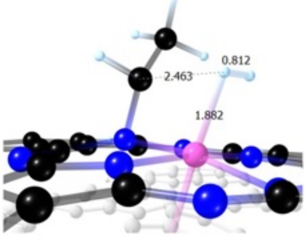

5’

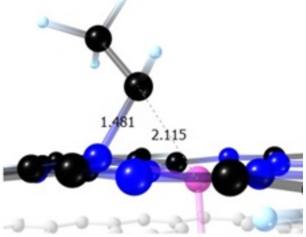

5

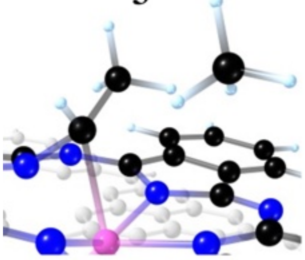

6

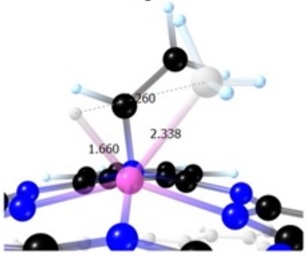

7

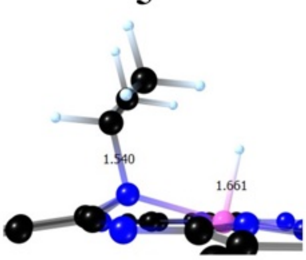

8

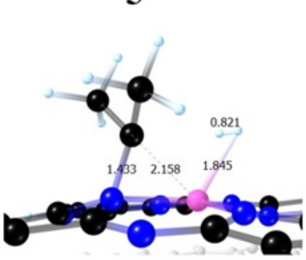

9’

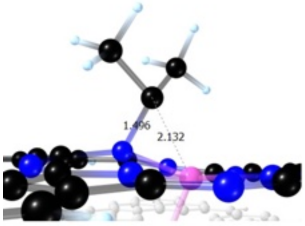

9

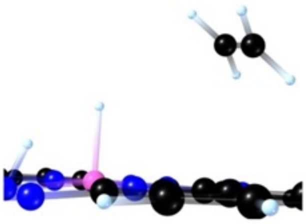

10

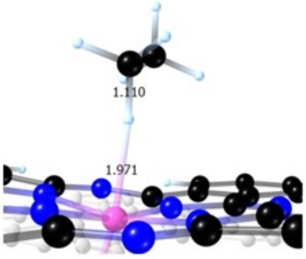

11

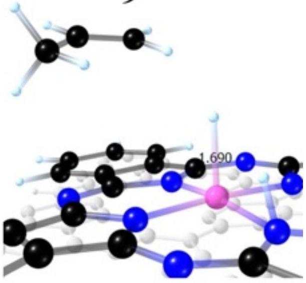

12

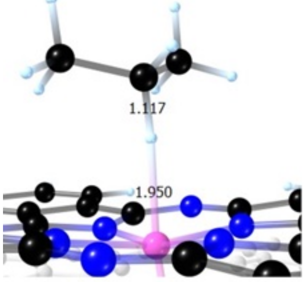

13

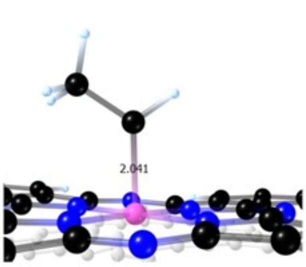

14

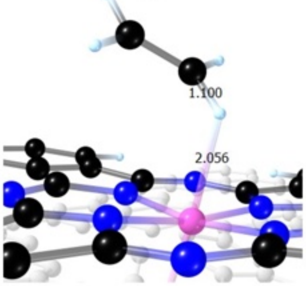

15

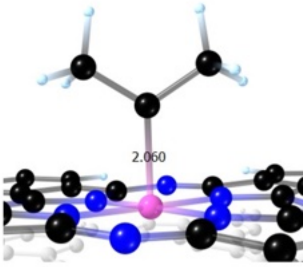

16

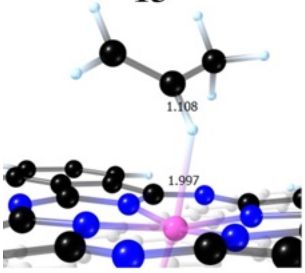

17

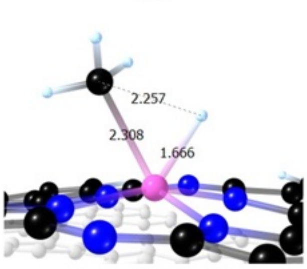

18

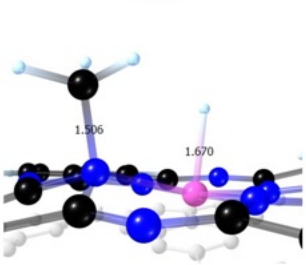

19

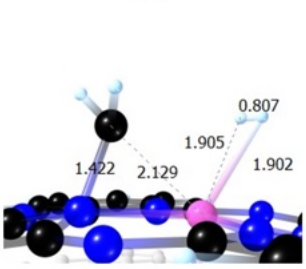

20

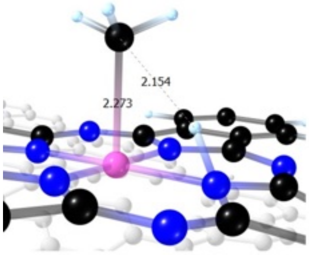

21

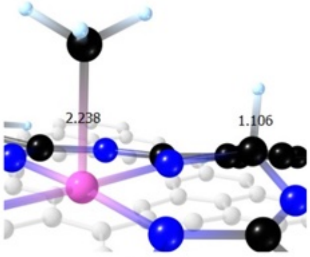

22

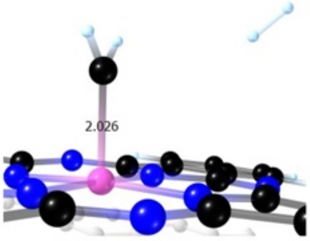

23’

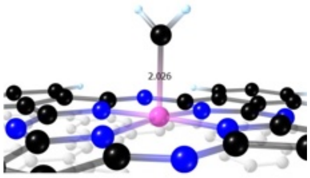

23

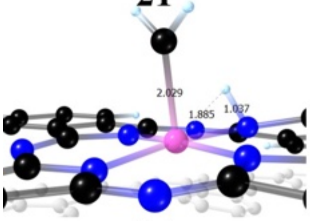

24

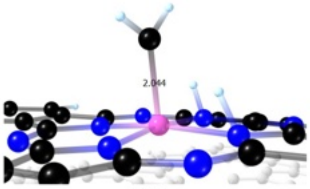

25

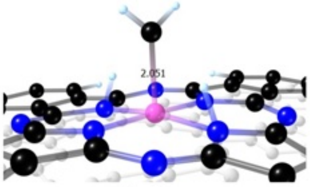

26

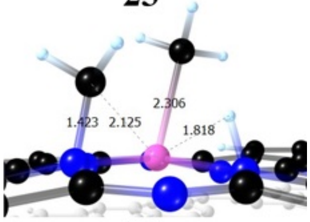

27

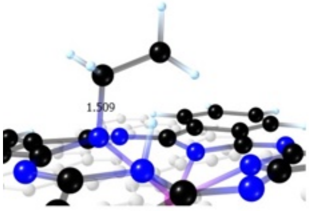

28

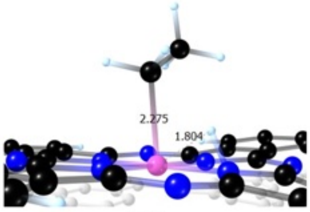

29

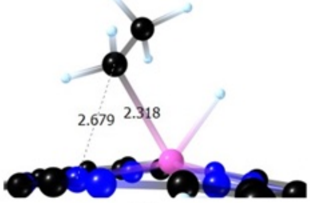

30

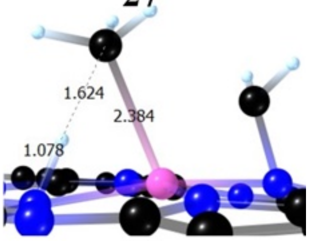

31

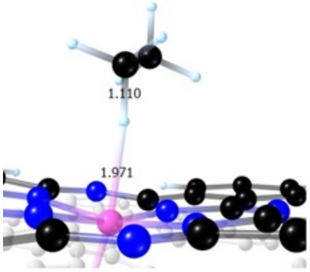

32

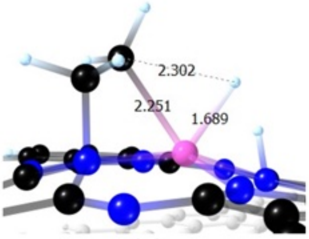

33

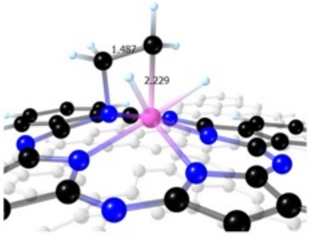

34

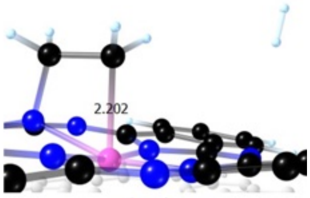

35’

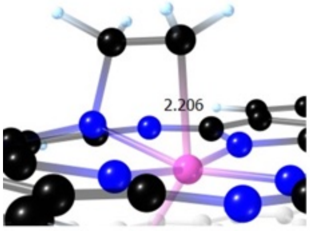

35

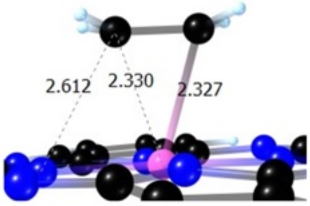

36

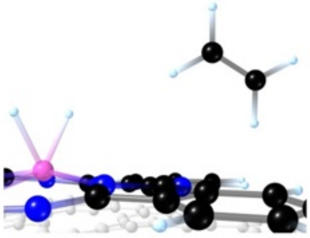

37

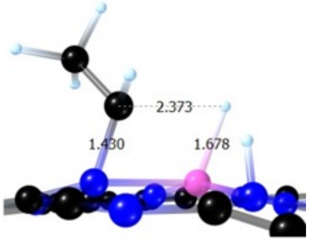

38

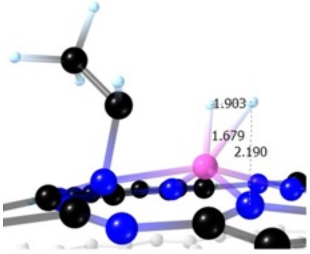

39

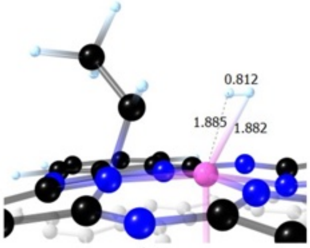

40

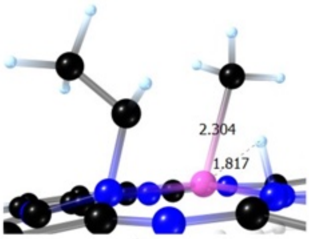

41

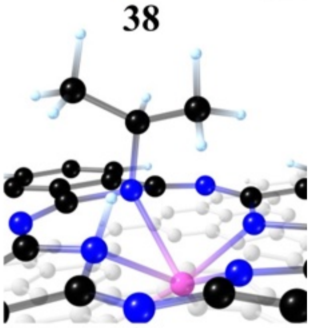

42

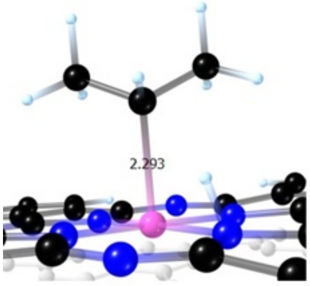

43

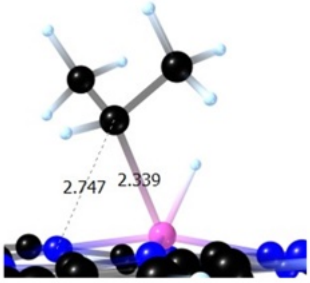

44

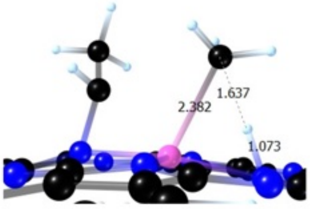

45

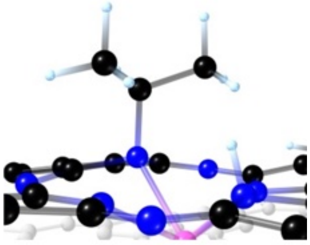

46

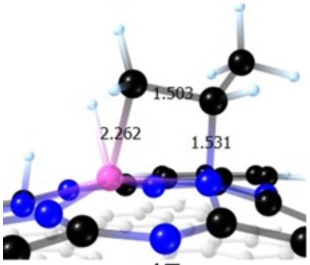

47

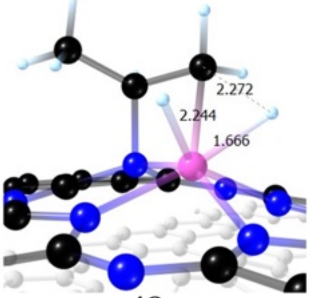

48

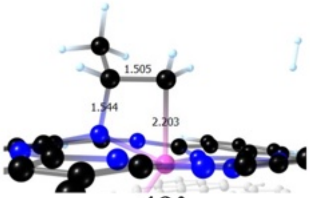

49’

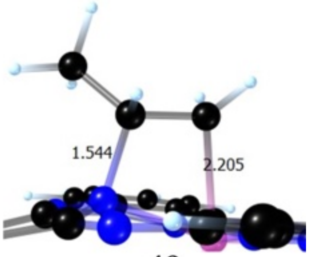

49

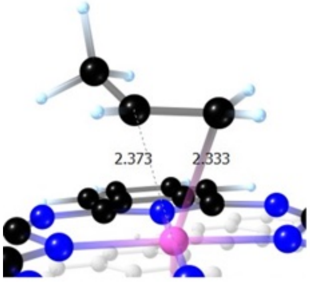

50

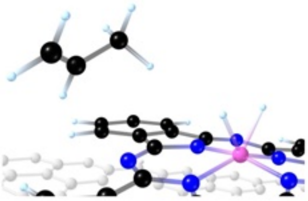

51

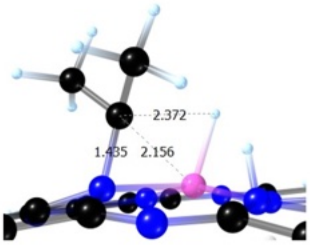

52

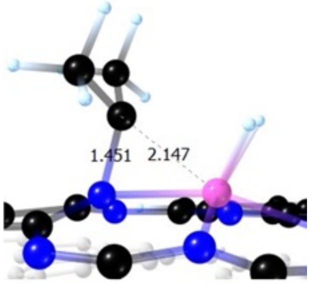

53’

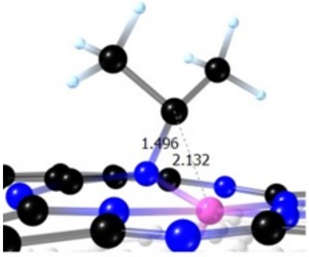

53

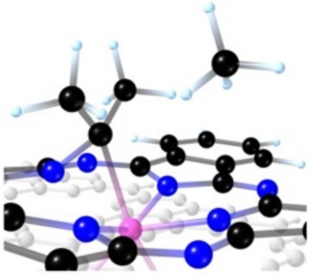

54

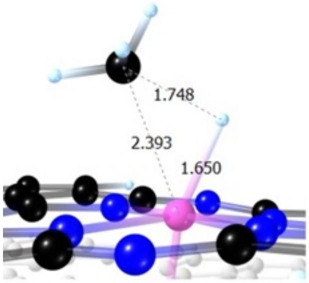

$\mathbf{Ts_{0-18}}$

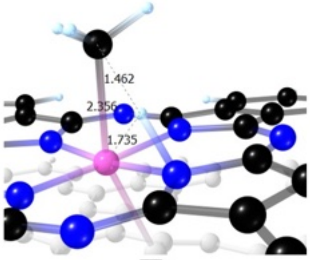

$\mathbf{Ts_{0-21}}$

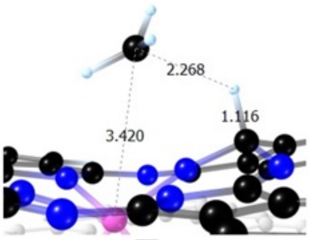

$\mathbf{Ts_{0-22}}$

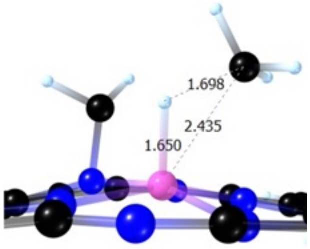

$\mathbf{Ts_{2-3}}$

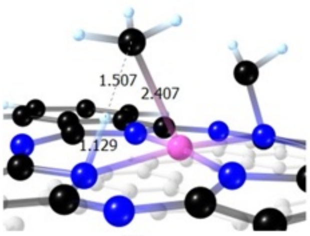

$\mathbf{Ts_{2-31}}$

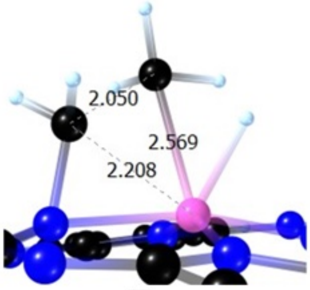

$\mathbf{Ts_{3-4}}$

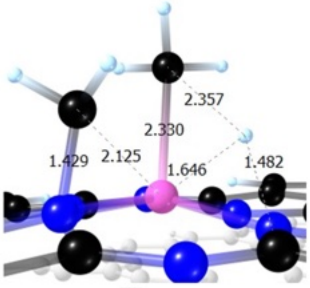

$\mathbf{Ts_{3-27}}$

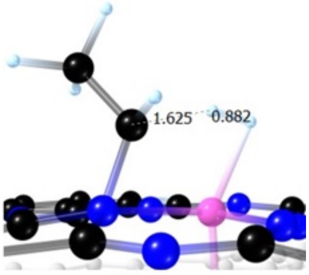

$\mathbf{Ts_{4-5}}$

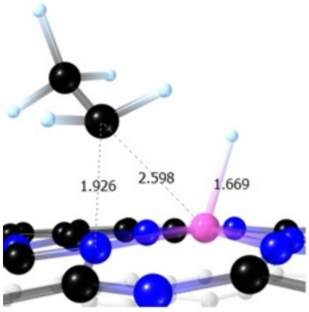

$\mathbf{Ts_{4-30}}$

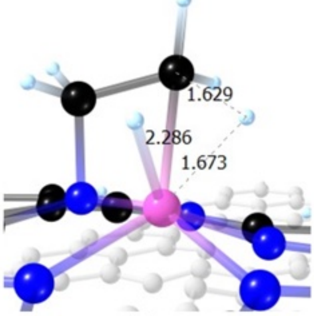

$\mathbf{Ts_{4-34}}$

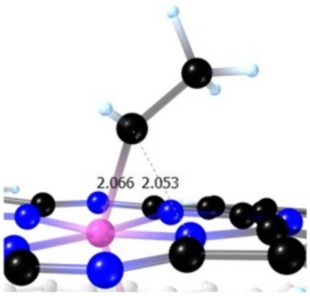

$\mathbf{Ts_{5-14}}$

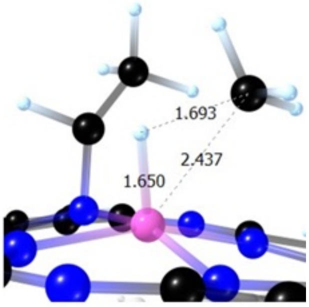

$\mathbf{Ts_{6-7}}$

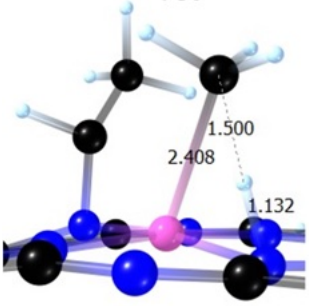

$\mathbf{Ts_{6-45}}$

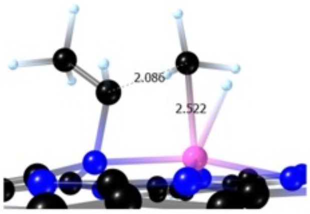

$\mathbf{Ts_{7-8}}$

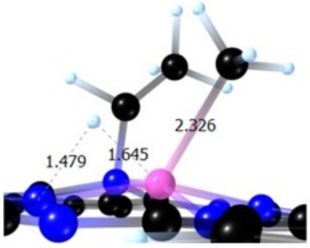

$\mathbf{Ts_{7-13}}$

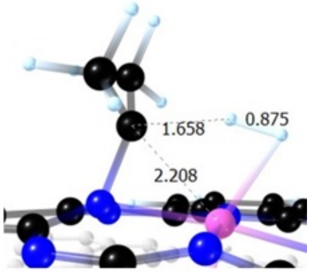

$\mathbf{Ts_{8-9}}$

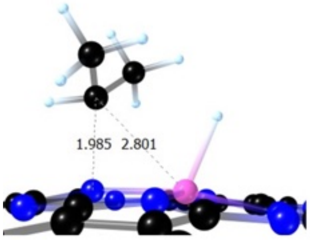

$\mathbf{Ts_{8-44}}$

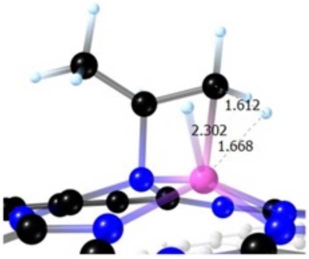

$\mathbf{Ts_{8-48}}$

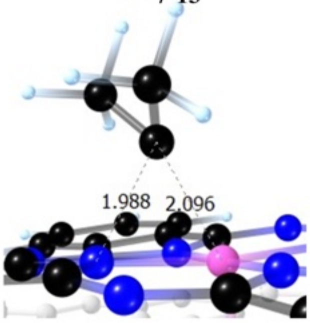

$\mathbf{Ts_{9-16}}$

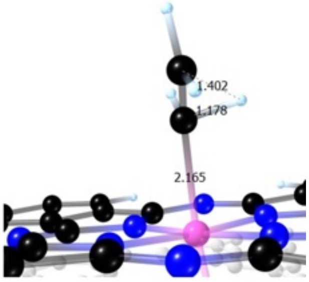

$\mathbf{Ts_{14-45}}$

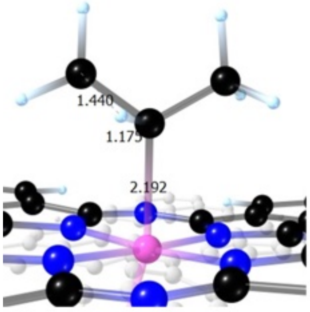

Ts_{16-17}

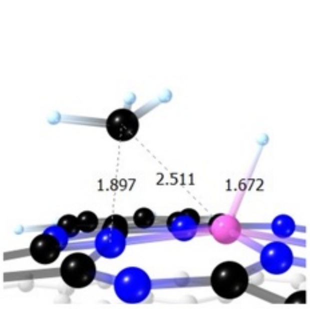

Ts_{18-19}

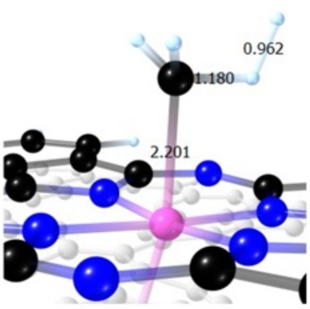

Ts_{18-23}

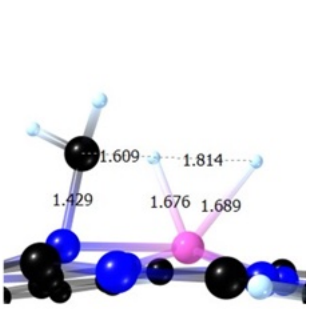

Ts_{19-20}

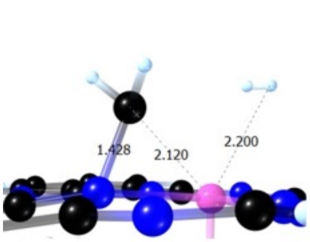

Ts_{20-1}

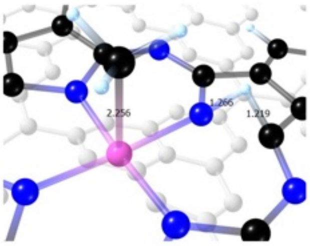
Ts_{21-22}

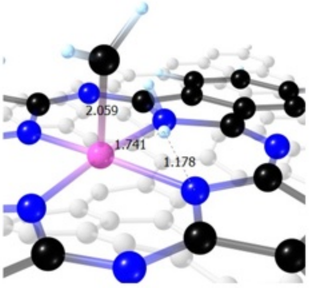

Ts_{21-25}

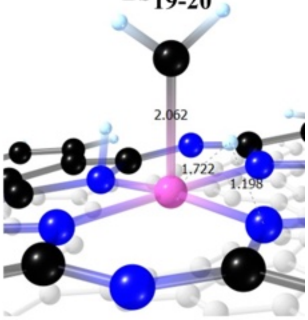

Ts_{21-26}

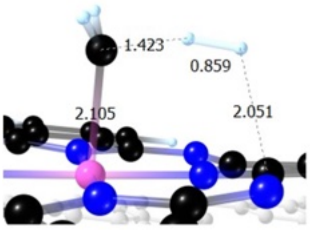

Ts_{22-23}

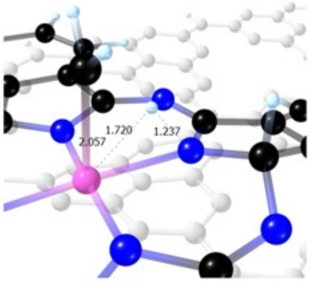
Ts_{22-24}

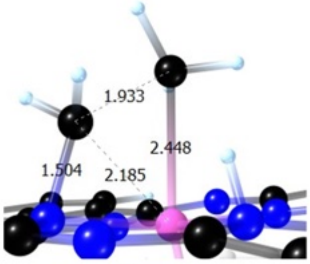

Ts_{27-28}

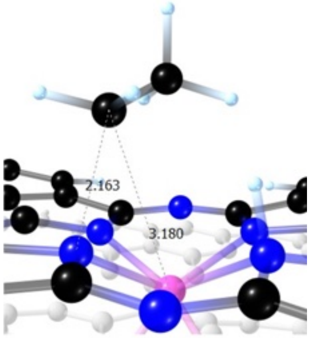

Ts_{28-29}

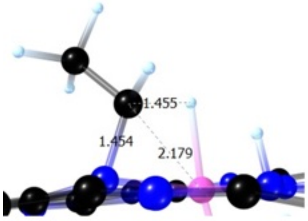

Ts_{28-38}

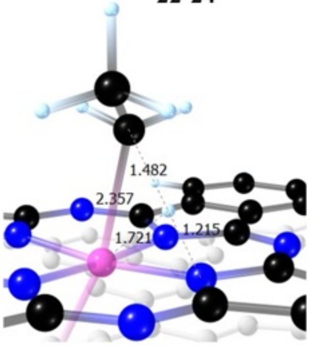

Ts_{29-11}

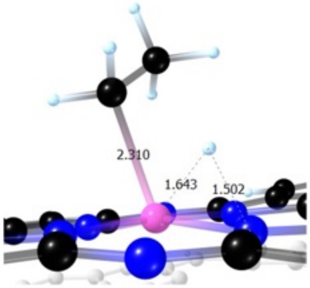

Ts_{29-30}

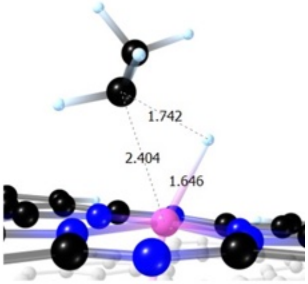

Ts_{30-11}

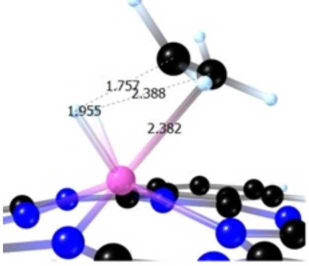

Ts_{30-37}

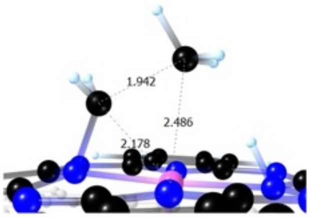

Ts_{31-32}

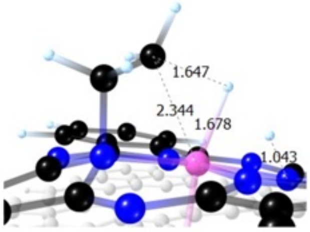

Ts_{32-33}

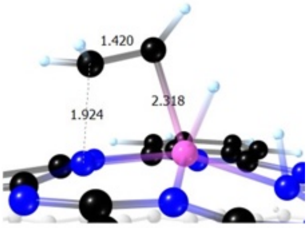

Ts_{33-10}

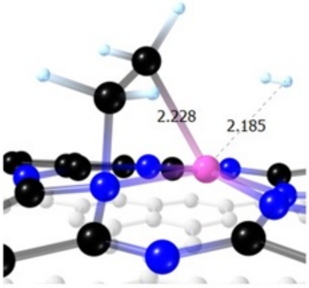

Ts_{34-35}

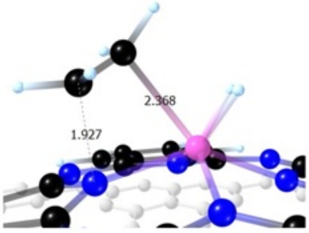

Ts_{34-37}

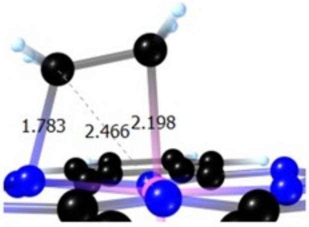

Ts_{35-36}

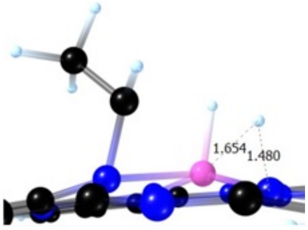

Ts_{38-39}

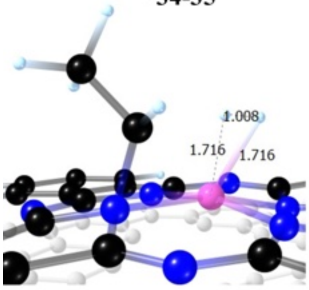

Ts_{39-40}

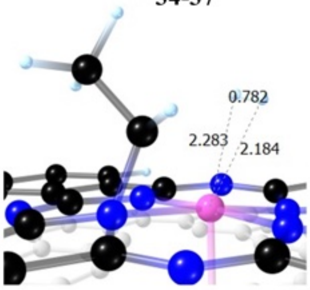

Ts_{40-5}

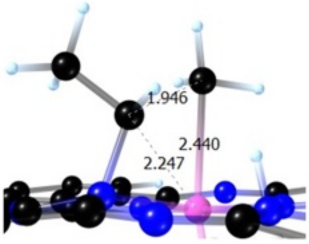

Ts_{41-42}

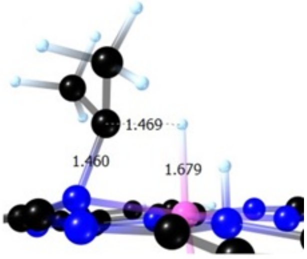

Ts_{42-52}

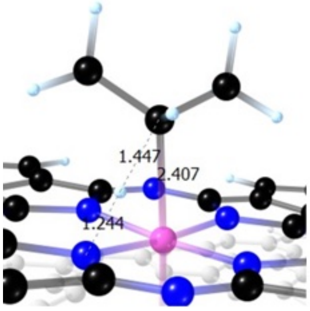

Ts_{43-13}

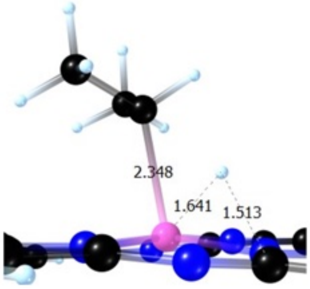

Ts_{43-44}

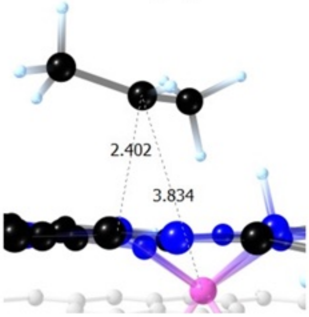

Ts_{42-43}

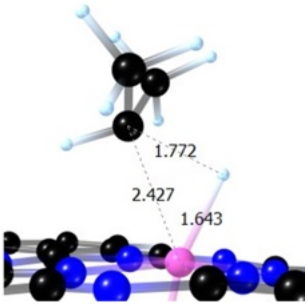

Ts_{44-13}

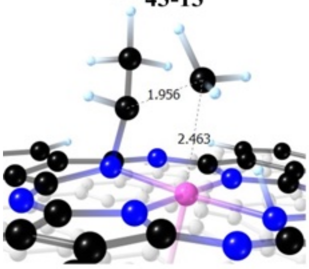

Ts_{46-47}

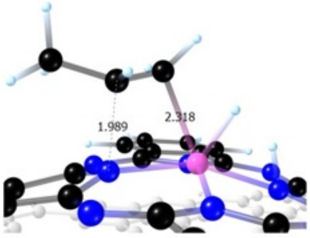

Ts_{47-12}

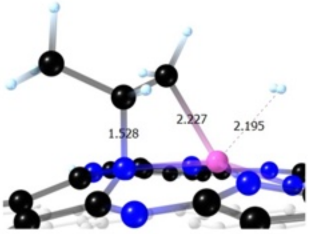

Ts_{48-49}

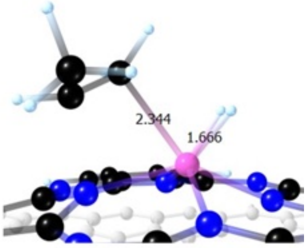

Ts_{48-51}

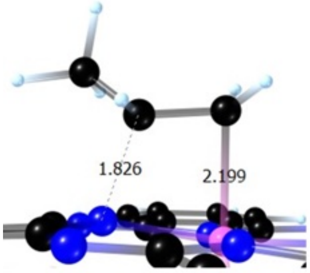

Ts_{49-50}

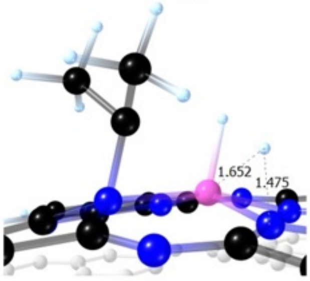

Ts_{52-53}

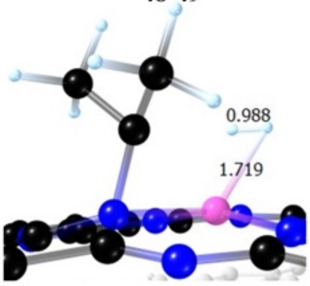

Ts_{53-9}

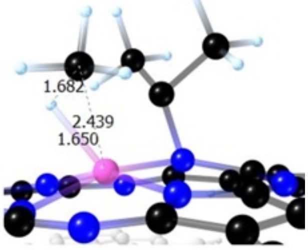

Ts_{53-9}

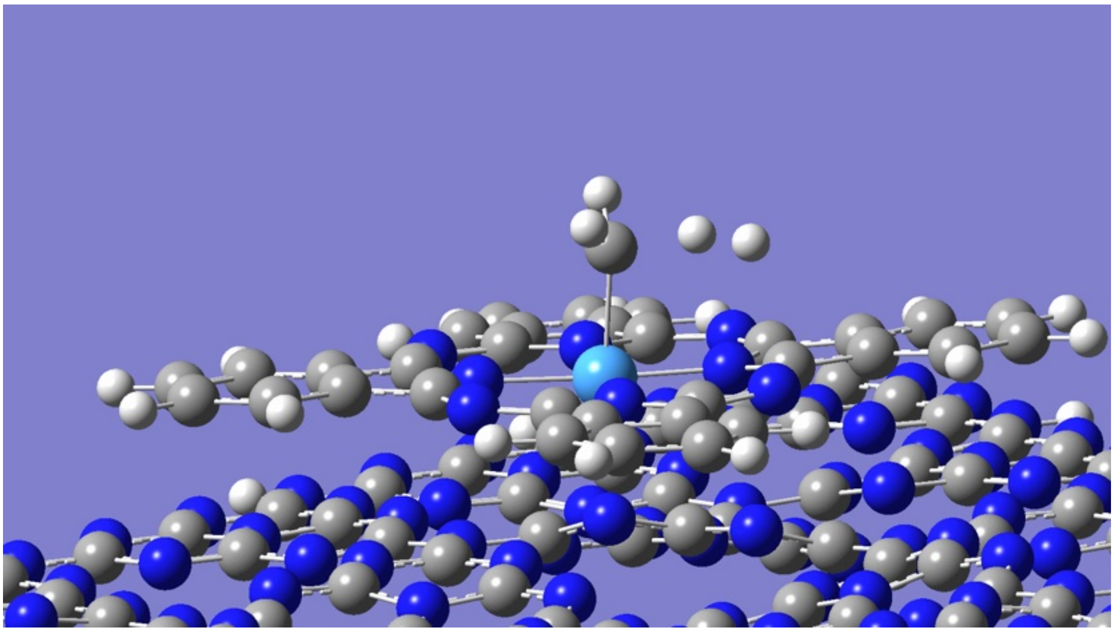

Figure S15 The key TS for Mvk-like mechanism.

The process proceeding via the Mvk-like mechanism has to overcome a large energy barrier (see Figure S15 for the TS structure) (a gap with 220 kJ/mol), thus making it inferior.

Reference

1. Bannwarth, C.; Ehlert, S.; Grimme, S. GFN2-xTB-An Accurate and Broadly Parametriz ed Self-Consistent Tight-Binding Quantum Chemical Method with Multipole Electrostati cs and Density-Dependent Dispersion Contributions. *J. Chem. Theory Comput.* **2019,** *15* (3), 1652-1671.
2. Grimme, S.; Bannwarth, C.; Shushkov, P. A Robust and Accurate Tight-Binding Quantu m Chemical Method for Structures, Vibrational Frequencies, and Noncovalent Interaction s of Large Molecular Systems Parametrized for All spd-Block Elements (Z=1-86). *J. C hem. Theory Comput.* **2017,** *13* (5), 1989-2009.
3. Frisch, M. J.; Trucks, G. W.; Schlegel, H. B.; Scuseria, G. E.; Robb, M. A.; Cheesem an, J. R.; Scalmani, G.; Barone, V.; Mennucci, B.; Petersson, G. A.; Nakatsuji, H.; Car icato, M.; Li, X.; Hratchian, H. P.; Izmaylov, A. F.; Bloino, J.; Zheng, G.; Sonnenberg, J. L.; Hada, M.; Ehara, M.; Toyota, K.; Fukuda, R.; Hasegawa, J.; Ishida, M.; Nakaji ma, T.; Honda, Y.; Kitao, O.; Nakai, H.; Vreven, T.; Montgomery, J. A.; Jr.; Peralta, J. E.; Ogliaro, F.; Bearpark, M.; Heyd, J. J.; Brothers, E.; Kudin, K. N.; Staroverov, V. N.; Kobayashi, R.; Normand, J.; Raghavachari, K.; Rendell, A.; Burant, J. C.; Iyengar, S. S.; Tomasi, J.; Cossi, M.; Rega, N.; Millam, J. M.; Klene, M.; Knox, J. E.; Cross, J. B.; Bakken, V.; Adamo, C.; Jaramillo, J.; Gomperts, R.; Stratmann, R. E.; Yazyev, O.; Austin, A. J.; Cammi, R.; Pomelli, C.; Ochterski, J. W.; Martin, R. L.; Morokuma, K.; Zakrzewski, V. G.; Voth, G. A.; Salvador, P.; Dannenberg, J. J.; Dapprich, S.; Dani els, A. D.; Farkas, O.; Foresman, J. B.; Ortiz, J. V.; Cioslowski, J.; Fox, D. J., Gaussi

an 09, Revision D.01, Gaussian, Inc., Wallingford CT, 2009.
4. Tian Lu, gau_xtb: A Gaussian interface for xtb code, http://sobereva.com/soft/gau_xtb.
5. Dean, J. A., *Lange's Handbook of Chemistry*. 2010; Vol. 5, p 687-688.
